# Comparative Analysis of Surrogate Insulin Resistance Indexes in Cardiometabolic Multimorbidity: A Cross-Sectional Study

**DOI:** 10.31083/RCM46005

**Published:** 2026-06-23

**Authors:** Jinyan Lei, Yuansong Zhuang, Xinlong Zhao, Yuxiong Chen, Siqi Tang, Yitao Han, Yakun Zhao, Yanbo Liu, Zhongjie Fan

**Affiliations:** ^1^Department of Cardiology, Peking Union Medical College Hospital, Chinese Academy of Medical Sciences&Peking Union Medical College, 100000 Beijing, China; ^2^Department of Internal Medicine, Peking Union Medical College Hospital, Chinese Academy of Medical Sciences&Peking Union Medical College, 100000 Beijing, China; ^3^Department of Healthcare, Peking Union Medical College Hospital, Chinese Academy of Medical Sciences&Peking Union Medical College, 100000 Beijing, China

**Keywords:** multimorbidity, cardiovascular diseases, insulin resistance, cross-sectional studies

## Abstract

**Background:**

Cardiometabolic multimorbidity (CMM), defined as the coexistence of two or more cardiometabolic diseases, poses an increasing challenge to global health. Although various surrogate indexes of insulin resistance (IR) exist, current evidence lacks a systematic comparison of the related strength of association with CMM. Therefore, this study aimed to systematically evaluate 12 IR surrogates to determine the associated discriminative ability for detecting CMM.

**Methods:**

The analytical cohort comprised 9756 eligible participants from the National Health and Nutrition Examination Survey (NHANES) conducted between 1999 and 2020. The 12 IR surrogates were examined using multivariable logistic regression to assess the related associations with CMM. Nonlinear relationships were examined with restricted cubic splines, and discriminatory power was evaluated through the area under the curve (AUC) from receiver operating characteristic (ROC) analysis.

**Results:**

Among 9756 eligible participants, 4383 (44.9%) were identified with CMM. Multivariable logistic regression-adjusted models revealed that odds ratios (ORs) for CMM progressively increased with ascending quartiles of all IR surrogates. After full adjustment, the ORs of homeostatic model assessment of insulin resistance (HOMA-IR) were as follows: Q2: OR = 1.78, 95% confidence interval (CI): 1.48–2.14; Q3: OR = 3.60, 95% CI: 2.82–4.60; Q4: OR = 8.80, 95% CI: 7.31–10.60. For triglyceride-glucose (TyG)-a body shape index (TyG-ABSI), the corresponding ORs were: Q2: OR = 2.51, 95% CI: 2.04–3.11; Q3: OR = 4.93, 95% CI: 3.98–6.10; Q4: OR = 11.02, 95% CI: 8.86–13.71. Nonlinear associations were observed for 11 of the 12 IR indexes, whereas TyG-ABSI exhibited linear dose–response relationships. TyG-ABSI outperformed other markers in the ROC analyses with an AUC of 0.764. These findings remained robust across multiple sensitivity analyses.

**Conclusions:**

TyG-ABSI is a reliable and cost-effective indicator associated with CMM risk, supporting the subsequent utility in distinguishing higher-risk individuals.

## 1. Introduction

Global population aging has made multimorbidity a critical public health issue [1]. Among various comorbidity patterns, cardiometabolic multimorbidity (CMM)—the coexistence of two or more cardiometabolic diseases (CMDs), such as coronary artery disease (CAD), diabetes, stroke, hypertension, or dyslipidemia—is a high-priority concern, particularly due to its prevalence and poor prognosis. Epidemiological evidence indicates that individuals with CMM have a life expectancy shortened by 12–15 years at age 60, with all-cause mortality 3.7–6.9 times higher compared to those without CMDs. CMM is also strongly associated with adverse outcomes, including physical disability, dementia, cognitive decline, and depression [2,3].

Insulin resistance (IR) is a key pathological mechanism underlying CMDs. IR contributes not only to metabolic disorders such as diabetes and atherosclerotic cardiovascular disease (ASCVD), but also independently predicts major adverse cardiovascular events (MACEs) [4]. IR drives the progression of CMM through multiple mechanisms, including dysregulated lipid metabolism, impaired fibrinolysis, and activation of the renin-angiotensin-aldosterone system [5,6]. Although early detection of IR is crucial for CMM prevention, current assessment methods remain limited: the gold-standard hyperinsulinemic-euglycemic clamp technique is invasive and costly, while the homeostasis model assessment of IR (HOMA-IR), though widely recognized, relies on fasting insulin measurement, restricting its use in resource-limited settings [7].

In recent years, various surrogate IR indexes based on conventional biomarkers and anthropometric parameters have been rapidly developed, including the triglyceride-glucose (TyG) index and its derivatives, lipid accumulation product (LAP), visceral adiposity index (VAI), and metabolic score for IR (METS-IR) [8,9]. Previous studies have identified associations between specific surrogate markers and the risk of CMM. However, current evidence remains fragmented, with most studies focusing on only one or a few IR markers [9,10,11,12,13]. A comprehensive comparison is still lacking regarding the associative strength of different IR surrogates with CMM risk, population-specific thresholds, and clinical applicability. Furthermore, no consensus has been reached on how to select cost-effective and accessible screening indicators tailored to the characteristics of specific populations.

This study incorporates 12 commonly used surrogate IR indexes, including LAP, VAI, cardiometabolic index (CMI), atherogenic index of plasma (AIP), triglyceride to high-density lipoprotein cholesterol ratio (TG/HDL-C), METS-IR, HOMA-IR, TyG, and its derivatives (TyG-body mass index [TyG-BMI], TyG-waist circumference [TyG-WC], TyG-waist-to-height ratio [TyG-WHtR], and TyG-a body shape index [TyG-ABSI]). Utilizing nationally representative data from NHANES within a cross-sectional framework, we aimed to: (1) examine the dose-response relationship of each index with CMM prevalence; (2) compare the discriminatory capacity of multiple IR surrogates in identifying CMM. By evaluating these indexes under a unified framework, this study seeks to inform the development of evidence-based, resource-tailored screening strategies for CMM.

## 2. Methods

### 2.1 Study Design and Population

As a nationally representative survey, NHANES collects data on health and nutritional status using a multistage stratified probability sampling design in the United States. All participants provided written informed consent. Further details are available on the official website of the Centers for Disease Control and Prevention (CDC) (https://www.cdc.gov/nchs/).

**Fig. 1.**
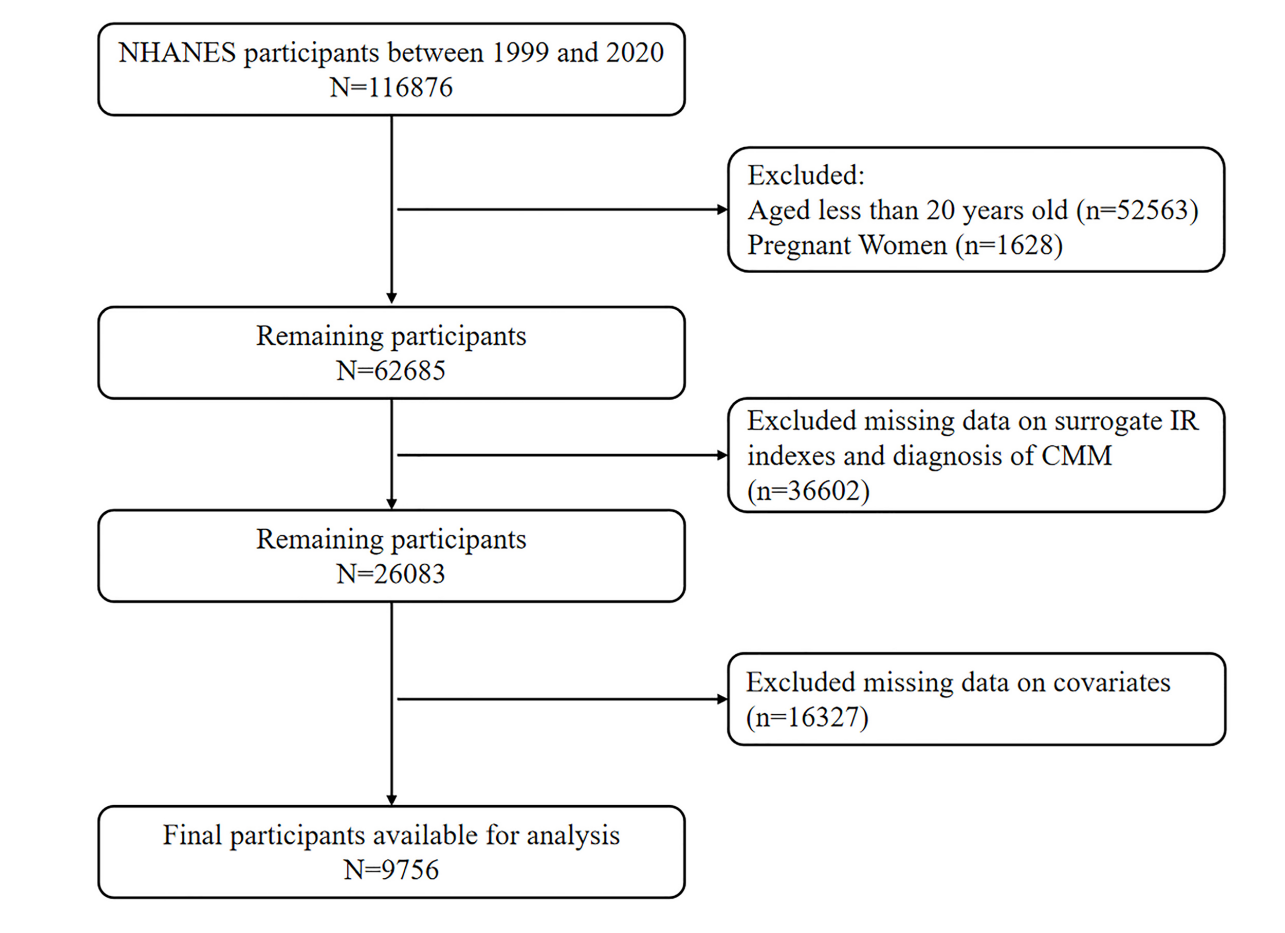
**Flow diagram of participant recruitment and screening**. NHANES, National Health and Nutrition Examination Survey; IR, insulin resistance; CMM, cardiometabolic multimorbidity.

A total of 116,876 individuals from the NHANES dataset spanning 1999 to 2020 were initially considered for inclusion in the study. The analytical population was derived by systematically excluding: (1) minors aged <20 years (n = 52,563); (2) pregnant individuals (n = 1628); (3) individuals with incomplete data for surrogate IR indexes or CMM diagnosis (n = 36,602); (4) individuals with missing covariate data, including demographic information and tobacco/alcohol use data (n = 16,327). Ultimately, 9756 participants were enrolled in our study (Fig. 1).

### 2.2 Evaluation of Different Surrogate IR Indexes

The calculation formulas of all surrogate IR indexes in our study are provided in Table 1 [9,12,14,15,16,17]. Blood specimens were collected after a minimum 8-hour fasting period for quantitative analysis of fasting plasma glucose, serum insulin, TG, and HDL-C concentrations. The measurement methods and quality control procedures of the laboratory indicators and physical parameters used in the calculation formulas are documented on the CDC website.

**Table 1.** **Calculation formulas for IR surrogates**.

IR surrogates	Formula used for derivation
LAP [14]	Men: LAP = [WC (cm) – 65] × [TG (mmol/L)]
	Women: LAP = [WC (cm) – 58] × [TG (mmol/L)]
VAI [12]	Men: VAI = WC (cm) / [39.68 + 1.88 × BMI (kg/m^2^)] × [TG (mmol/L) / 1.03] × [1.31 / HDL-C (mmol/L)
	Women: VAI = WC (cm) / [36.58 + 1.89 × BMI (kg/m^2^)] × [TG (mmol/L) / 0.81] × [1.52 / HDL-C (mmol/L)]
CMI [16]	CMI = [TG (mg/dL) / HDL-C (mg/dL)] × [WC (cm) / height (cm)]
AIP [14]	AIP = lg [TG (mmol/L) / HDL-C (mmol/L)]
TG/HDL-C [17]	TG/HDL-C = TG (mg/dL) / HDL-C (mg/dL)
METS-IR [9]	METS-IR = ln [2 × fasting plasma glucose (FPG) (mg/dL) + TG (mg/dL)] × BMI (kg/m^2^)/ln [HDL-C (mg/dL)]
HOMA-IR [9]	HOMA-IR = (fasting glucose [mmol/L] × fasting insulin [µU/mL]) / 22.5
TyG [14]	TyG = In [TG (mg/dL) × fasting glucose (mg/dL) / 2]
TyG-BMI [14]	TyG-BMI = TyG × BMI (kg/m^2^)
TyG-WC [14]	TyG-WC = TyG × WC (cm)
TyG-WHtR [9]	TyG-WHtR = TyG × [WC (cm) / height (cm)]
TyG-ABSI [15]	TyG-ABSI = TyG × [WC (cm) / (BMI^2/3^ × height^1/2^ (cm))]

LAP, lipid accumulation product; VAI, visceral adiposity index; CMI, cardiometabolic index; AIP, atherogenic index of plasma; TG/HDL-C, triglyceride to high-density lipoprotein cholesterol ratio; METS-IR, metabolic score for insulin resistance; HOMA-IR, homeostatic model assessment of insulin resistance; TyG, triglyceride-glucose; TyG-BMI, TyG-body mass index; TyG-WC, TyG-waist circumference; TyG-WHtR, TyG-waist-to-height ratio; TyG-ABSI, TyG-a body shape index; BMI, body mass index; TG, triglyceride; WC, waist circumference; IR, insulin resistance.

### 2.3 Assessment of CMM

CMM was defined as the coexistence of ≥2 CMDs, including hypertension (HTN), diabetes mellitus (DM), CAD, stroke, and hyperlipidemia. HTN was established through self-reported physician diagnosis, systolic blood pressure (SBP) ≥140 mmHg, diastolic blood pressure (DBP) ≥90 mmHg, or current use of antihypertensive agents. DM diagnosis required meeting one or more criteria: hemoglobin A1c (HbA1c) ≥6.5%; fasting plasma glucose (FPG) ≥7.0 mmol/L; 2-hour oral glucose tolerance test (OGTT 2hPG) ≥11.1 mmol/L; self-reported physician diagnosis; or current use of anti-diabetic therapy. CAD and stroke were identified through self-reported physician diagnoses. Hyperlipidemia classification relied on the National Cholesterol Education Program Adult Treatment Panel III (NCEP-ATP III) guidelines. Participants were classified as having hyperlipidemia if they met any of the following criteria: total cholesterol (TC) ≥200 mg/dL; TG ≥150 mg/dL; low-density lipoprotein cholesterol (LDL-C) ≥130 mg/dL; HDL-C <40 mg/dL in men or <50 mg/dL in women; or current use of lipid-lowering medications [18]. Definitions of self-reported outcomes and further operational details are provided in **Supplementary Table 1**.

### 2.4 Covariates

Covariates included age, sex, race, educational levels, marital status, poverty income ratio (PIR), BMI, as well as tobacco use and alcohol use. Individuals were designated ‘never smokers’ if they reported a lifetime consumption of fewer than 100 cigarettes. Those who had smoked 100 or more cigarettes in their lifetime, including both past and current smokers, were classified as smokers. Similarly, alcohol use was categorized as follows: ‘never drinkers’ were those who had consumed fewer than 12 alcoholic drinks in their lifetime, while ‘drinkers’ included those who had consumed 12 or more alcoholic drinks in their lifetime or were current drinkers.

### 2.5 Statistical Analysis

To accurately account for the complex, multistage sampling design of NHANES, all analyses were conducted in accordance with the guidelines established by the CDC. Sampling weights, primary sampling units (SDMVPSU), and stratification variables (SDMVSTRA) were incorporated throughout, using the survey package in R 4.4.3 (R Foundation for Statistical Computing, Vienna, Austria). In accordance with the CDC recommendations for multi-cycle analysis, sampling weights were assigned as follows: participants from 1999–2002 were assigned the WTMEC4YR multiplied by 2/11.625; those from 2003–2016 were assigned the corresponding WTMEC2YR multiplied by 1/11.625; and participants from 2017–2020, during which data collection spanned 39 months due to the COVID-19 pandemic, were assigned the WTMECPRP multiplied by 1.625/11.625. The latter adjustment factor of 1.625 (i.e., 39/24) follows the standardization guidance provided by the National Center for Health Statistics (NCHS). Continuous variables were expressed as weighted means with standard errors, and categorical variables were summarized as absolute frequencies with survey-weighted percentages. Weighted *t*-tests were applied to compare continuous variables, while chi-square tests were used for categorical comparisons. Multivariate logistic regression analysis served to evaluate the association between surrogate IR indexes (categorized into quartiles) and CMM. Outcomes were presented as odds ratios (ORs) accompanied by 95% confidence intervals (CIs). These models were adjusted for age, sex, race, PIR, marital status, and education levels, tobacco use and alcohol use. Dose-response relationships were explored by treating IR surrogate markers as continuous variables, and assessing CMM risk per standard deviation (SD) increase in each index. A *p*-value for trend was calculated to assess the trend of ORs across the quartiles of IR surrogates.

Restricted cubic splines (RCS) were employed to examine potential nonlinear relationships between surrogate IR indicators and CMM. All RCS analyses were configured with four knots placed at the 5th, 35th, 65th, and 95th percentiles of the exposure variables. In addition, we conducted stratified subgroup analyses (by age, sex, race, BMI, tobacco, and alcohol use), presenting these findings with forest plots. In the subgroup analyses, we applied a linear scaling of ×10 to TyG-ABSI due to its extremely small original values. Calculating the odds ratio per 1-unit increase on the original scale would correspond to an unreasonably large and physiologically implausible change, resulting in artificially inflated and difficult-to-interpret ORs. Scaling TyG-ABSI by 10-fold makes the effect estimates more intuitive and readable while preserving statistical significance and the direction of association, without altering the results of the interaction tests. The discriminative capacity of each IR surrogate for CMM was assessed by generating receiver operating characteristic (ROC) curves and calculating the area under these curves (AUC). ROC curves and AUC values reported in this study were derived from the raw values of the IR surrogates without covariate adjustment. DeLong test was utilized to compare the AUCs between different ROC curves. The primary inferential analyses of association are based on survey-weighted logistic regression models, which account for the complex survey design and provide nationally representative estimates. As secondary descriptive assessments of cross-sectional discrimination within the analytic sample, the discriminative capacity of each IR surrogate for CMM was evaluated by generating ROC curves and calculating AUC. All data processing and statistical tests were conducted with R 4.4.3. Statistical significance was established at a two-sided *p*-value < 0.05.

## 3. Results

### 3.1 Participants Characteristics

Table 2 displays the characteristics of the study population. Among the 9756 participants, 4383 (44.9%) were classified into the CMM group. Significant differences were observed between CMM and Non-CMM groups. The CMM group was older and had a higher proportion of females (61.04% vs 56.59%; *p *= 0.003) compared to Non-CMM participants. Socioeconomic differences were particularly evident in the CMM group, including lower educational levels (20.82% vs 12.64% with less than high school education; *p *< 0.001), higher poverty rates (29.90% vs 24.28% in the lowest PIR category; *p *< 0.001), and a greater proportion of widowed individuals (18.93% vs 8.17%; *p *< 0.001). Tobacco use was also higher in the CMM group (41.04% vs 34.20%;* p *< 0.001), while alcohol consumption was lower (74.03% vs 78.88%; *p *< 0.001). Notably, all IR surrogates were elevated in the CMM group (*p *< 0.001).

**Table 2.** Comparison of participant characteristics between CMM and Non-CMM groups from NHANES 1999–2020^*^.

Characteristics		Total (n = 9756)	Non-CMM (n = 5373)	CMM (n = 4383)	*p* value
Age, years		49.09 (0.37)	43.55 (0.36)	58.19 (0.43)	<0.001
Sex, n (%)					0.003
	Male	3911 (41.73)	2222 (43.41)	1689 (38.96)	
	Female	5845 (58.27)	3151 (56.59)	2694 (61.04)	
Race, n (%)					<0.001
	Mexican American	1592 (8.80)	932 (9.89)	660 (7.02)	
	Non-Hispanic Black	2270 (11.89)	1179 (11.25)	1091 (12.95)	
	Non-Hispanic White	3678 (63.80)	1959 (63.21)	1719 (64.77)	
	Other Hispanic	866 (6.15)	473 (6.23)	393 (6.03)	
	Other Race	1350 (9.35)	830 (9.42)	520 (9.23)	
Education levels, n (%)					<0.001
	Lower than high school	2414 (15.74)	1083 (12.64)	1331 (20.82)	
	High school	2314 (26.06)	1218 (23.81)	1096 (29.76)	
	Higher than high school	5028 (58.20)	3072 (63.55)	1956 (49.42)	
Marital status, n (%)					<0.001
	Married/living with partner	5797 (62.87)	3267 (63.47)	2530 (61.88)	
	Divorced/separated	1483 (14.53)	847 (15.44)	636 (13.05)	
	Widowed	1441 (12.24)	503 (8.17)	938 (18.93)	
	Never married	1035 (10.35)	756 (12.92)	279 (6.14)	
PIR, n (%)					<0.001
	0–1.5	3568 (26.41)	1838 (24.28)	1730 (29.90)	
	1.5–3.5	3396 (33.16)	1843 (31.47)	1553 (35.93)	
	>3.5	2792 (40.43)	1692 (44.24)	1100 (34.17)	
BMI, n (%)					<0.001
	0–25	2668 (28.61)	1851 (35.44)	817 (17.40)	
	25–30	3114 (31.28)	1714 (31.92)	1400 (30.24)	
	>30	3974 (40.11)	1808 (32.65)	2166 (52.36)	
Tobacco use, n (%)					<0.001
	Yes	3447 (36.79)	1708 (34.20)	1739 (41.04)	
	No	6309 (63.21)	3665 (65.80)	2644 (58.96)	
Alcohol use, n (%)					<0.001
	Yes	7022 (77.05)	3926 (78.88)	3096 (74.03)	
	No	2734 (22.95)	1447 (21.12)	1287 (25.97)	
LAP		56.63 (0.96)	42.35 (0.84)	80.05 (1.77)	<0.001
VAI		1.99 (0.03)	1.57 (0.03)	2.69 (0.06)	<0.001
CMI		1.67 (0.03)	1.28 (0.03)	2.30 (0.06)	<0.001
AIP		–0.07 (0.01)	–0.16 (0.01)	0.07 (0.01)	<0.001
TG/HDL-C		2.69 (0.05)	2.17 (0.05)	3.54 (0.08)	<0.001
METS-IR		43.96 (0.22)	40.82 (0.23)	49.12 (0.36)	<0.001
HOMA-IR		3.96 (0.10)	2.61 (0.05)	6.19 (0.24)	<0.001
TyG		8.57 (0.01)	8.34 (0.01)	8.95 (0.02)	<0.001
TyG-BMI		254.60 (1.15)	236.52 (1.22)	284.28 (1.87)	<0.001
TyG-WC		860.11 (2.98)	803.32 (2.92)	953.30 (4.53)	<0.001
TyG-WHtR		5.16 (0.02)	4.79 (0.02)	5.75 (0.03)	<0.001
TyG-ABSI		0.70 (0.00)	0.67 (0.00)	0.74 (0.00)	<0.001
HTN, n (%)					<0.001
	Yes	4585 (40.64)	808 (12.80)	3777 (86.30)	
	No	5171 (59.36)	4565 (87.20)	606 (13.70)	
CAD, n (%)					<0.001
	Yes	779 (6.50)	40 (0.65)	739 (16.11)	
	No	8977 (93.50)	5333 (99.35)	3644 (83.89)	
DM, n (%)					<0.001
	Yes	2221 (17.10)	133 (1.82)	2088 (42.15)	
	No	7535 (82.90)	5240 (98.18)	2295 (57.85)	
Stroke, n (%)					<0.001
	Yes	438 (3.31)	12 (0.32)	426 (8.23)	
	No	9318 (96.69)	5361 (99.68)	3957 (91.77)	
Dyslipidemia, n (%)					<0.001
	Yes	6302 (63.31)	2615 (48.81)	3687 (87.09)	
	No	3454 (36.69)	2758 (51.19)	696 (12.91)	

^*^ Continuous variables were expressed as weighted means with standard errors, and categorical variables were summarized as absolute frequencies with survey-weighted percentages. PIR, poverty income ratio; HTN, hypertension; CAD, coronary artery disease; DM, diabetes mellitus.

### 3.2 Association Analysis: Various Surrogate IR Indexes and CMM

To explore the associations between different IR indicators and CMM, we conducted multivariable logistic regression analyses across three hierarchical models. As shown in Table 3, all IR surrogates indicated a progressive increase in CMM risk as quartiles rose, with statistical significance maintained throughout. Notably, among the indicators derived from TG, HDL-C, and anthropometric parameters (waist circumference (WC), height, BMI), the increase in risks from LAP quartiles Q3 to Q4 remained particularly pronounced after full adjustment. When calculated in a per-SD increase, changes in ORs for LAP and VAI from Model 1 to Model 2 were relatively modest (ΔOR <10%). In contrast, the ORs per-SD increase for CMI, AIP, and TG/HDL-C showed a significant increase following adjustment by Model 2, although these associations tended to converge following full adjustment.

**Table 3.** **Multivariable logistic regression assessing associations of different IR surrogates with CMM**.

		Model 1		Model 2		Model 3	
		OR (95% CI)	*p* value	OR (95% CI)	*p* value	OR (95% CI)	*p* value
LAP							
	Per-SD increase	2.99 (2.50–3.57)	<0.001	2.98 (2.50–3.54)	<0.001	2.97 (2.49–3.53)	<0.001
	Q1	Reference	-	Reference	-	Reference	-
	Q2	3.26 (3.00–4.38)	<0.001	2.85 (2.30–3.53)	<0.001	2.86 (2.30–3.55)	<0.001
	Q3	6.70 (5.54–8.11)	<0.001	5.37 (4.35–6.62)	<0.001	5.37 (4.35–6.63)	<0.001
	Q4	13.89 (11.57–16.67)	<0.001	13.28 (11.00–16.03)	<0.001	13.26 (10.94–16.06)	<0.001
	*p* for trend	<0.001		<0.001		<0.001	
VAI							
	Per-SD increase	2.41 (2.01–2.90)	<0.001	2.43 (2.01–2.94)	<0.001	2.41 (1.99–2.93)	<0.001
	Q1	Reference	-	Reference	-	Reference	-
	Q2	2.35 (1.91–2.90)	<0.001	2.17 (1.76–2.67)	<0.001	2.16 (1.75–2.67)	<0.001
	Q3	5.07 (4.22–6.10)	<0.001	4.95 (4.07–6.02)	<0.001	4.93 (4.05–6.00)	<0.001
	Q4	9.17 (7.58–11.09)	<0.001	9.87 (8.17–11.94)	<0.001	9.78 (8.06–11.87)	<0.001
	*p* for trend	<0.001		<0.001		<0.001	
CMI							
	Per-SD increase	2.49 (2.04–3.04)	<0.001	2.68 (2.14–3.34)	<0.001	2.66 (2.13–3.33)	<0.001
	Q1	Reference	-	Reference	-	Reference	-
	Q2	2.23 (1.82–2.73)	<0.001	1.92 (1.55–2.38)	<0.001	1.92 (1.54–2.38)	<0.001
	Q3	4.55 (3.69–5.61)	<0.001	4.33 (3.51–5.33)	<0.001	4.31 (3.50–5.31)	<0.001
	Q4	8.84 (7.20–10.85)	<0.001	10.58 (8.56–13.07)	<0.001	10.49 (8.44–13.05)	<0.001
	*p* for trend	<0.001		<0.001		<0.001	
AIP							
	Per-SD increase	2.10 (1.96–2.26)	<0.001	2.36 (2.19–2.55)	<0.001	2.35 (2.17–2.55)	<0.001
	Q1	Reference	-	Reference	-	Reference	-
	Q2	2.18 (1.78–2.66)	<0.001	2.11 (1.73–2.57)	<0.001	2.11 (1.73–2.57)	<0.001
	Q3	4.31 (3.49–5.32)	<0.001	4.50 (3.65–5.55)	<0.001	4.48 (3.63–5.53)	<0.001
	Q4	7.25 (6.02–8.73)	<0.001	9.38 (7.84–11.23)	<0.001	9.29 (7.74–11.16)	<0.001
	*p* for trend	<0.001		<0.001		<0.001	
TG/HDL-C							
	Per-SD increase	2.10 (1.74–2.52)	<0.001	2.28 (1.85–2.83)	<0.001	2.27 (1.83–2.82)	<0.001
	Q1	Reference	-	Reference	-	Reference	-
	Q2	2.21 (1.80–2.71)	<0.001	2.13 (1.75–2.61)	<0.001	2.13 (1.75–2.61)	<0.001
	Q3	4.33 (3.51–5.35)	<0.001	4.52 (3.66–5.57)	<0.001	4.50 (3.65–5.55)	<0.001
	Q4	7.35 (6.09–8.87)	<0.001	9.50 (7.92–11.40)	<0.001	9.42 (7.83–11.33)	<0.001
	*p* for trend	<0.001		<0.001		<0.001	
METS-IR							
	Per-SD increase	1.99 (1.86–2.12)	<0.001	2.38 (2.21–2.56)	<0.001	2.39 (2.22–2.57)	<0.001
	Q1	Reference	-	Reference	-	Reference	-
	Q2	2.24 (1.86–2.70)	<0.001	1.88 (1.57–2.27)	<0.001	1.89 (1.57–2.28)	<0.001
	Q3	3.10 (2.57–3.73)	<0.001	3.22 (2.60–3.98)	<0.001	3.24 (2.62–4.00)	<0.001
	Q4	6.13 (5.13–7.32)	<0.001	8.61 (7.05–10.53)	<0.001	8.75 (7.16–10.69)	<0.001
	*p* for trend	<0.001		<0.001		<0.001	
HOMA-IR							
	Per-SD increase	5.81 (4.71–7.16)	<0.001	6.85 (5.55–8.45)	<0.001	6.83 (5.53–8.44)	<0.001
	Q1	Reference	-	Reference	-	Reference	-
	Q2	1.87 (1.57–2.23)	<0.001	1.78 (1.48–2.14)	<0.001	1.78 (1.48–2.14)	<0.001
	Q3	3.52 (2.82–4.41)	<0.001	3.59 (2.82–4.58)	<0.001	3.60 (2.82–4.60)	<0.001
	Q4	7.16 (5.92–8.66)	<0.001	8.78 (7.30–10.56)	<0.001	8.80 (7.31–10.60)	<0.001
	*p* for trend	<0.001		<0.001		<0.001	
TyG							
	Per-SD increase	3.00 (2.74–3.28)	<0.001	3.02 (2.75–3.32)	<0.001	3.01 (2.73–3.31)	<0.001
	Q1	Reference	-	Reference	-	Reference	-
	Q2	3.30 (2.74–3.96)	<0.001	2.70 (2.23–3.28)	<0.001	2.70 (2.22–3.28)	<0.001
	Q3	6.58 (5.26–8.24)	<0.001	5.61 (4.41–7.14)	<0.001	5.59 (4.40–7.12)	<0.001
	Q4	16.27 (13.22–20.03)	<0.001	15.63 (12.61–19.38)	<0.001	15.48 (12.49–19.20)	<0.001
	*p *for trend	<0.001		<0.001		<0.001	
TyG-BMI							
	Per-SD increase	2.09 (1.96–2.23)	<0.001	2.36 (2.19–2.54)	<0.001	2.37 (2.20–2.55)	<0.001
	Q1	Reference	-	Reference	-	Reference	-
	Q2	2.78 (2.24–3.45)	<0.001	2.17 (1.74–2.72)	<0.001	2.19 (1.75–2.74)	<0.001
	Q3	4.10 (3.39–4.97)	<0.001	3.87 (3.07–4.87)	<0.001	3.89 (3.10–4.90)	<0.001
	Q4	7.61 (6.36–9.11)	<0.001	9.20 (7.49–11.31)	<0.001	9.33 (7.60–11.47)	<0.001
	*p* for trend	<0.001		<0.001		<0.001	
TyG-WC							
	Per-SD increase	2.56 (2.40–2.73)	<0.001	2.68 (2.49–2.88)	<0.001	2.69 (2.50–2.89)	<0.001
	Q1	Reference	-	Reference	-	Reference	-
	Q2	2.80 (2.23–3.51)	<0.001	2.17 (1.67–2.82)	<0.001	2.18 (1.68–2.84)	<0.001
	Q3	5.29 (4.35–6.43)	<0.001	4.41 (3.53–5.50)	<0.001	4.44 (3.55–5.55)	<0.001
	Q4	10.58 (8.53–13.11)	<0.001	10.58 (8.38–13.36)	<0.001	10.68 (8.46–13.50)	<0.001
	*p* for trend	<0.001		<0.001		<0.001	
TyG-WHtR							
	Per-SD increase	2.82 (2.63–3.03)	<0.001	2.69 (2.50–2.90)	<0.001	2.70 (2.51–2.91)	<0.001
	Q1	Reference	-	Reference	-	Reference	-
	Q2	3.62 (3.03–4.33)	<0.001	2.56 (2.08–3.15)	<0.001	2.57 (2.08–3.18)	<0.001
	Q3	6.50 (5.37–7.86)	<0.001	4.69 (3.83–5.74)	<0.001	4.71 (3.85–5.77)	<0.001
	Q4	14.88 (12.12–18.27)	<0.001	11.78 (9.47–14.66)	<0.001	11.85 (9.51–14.75)	<0.001
	*p* for trend	<0.001		<0.001		<0.001	
TyG-ABSI							
	Per-SD increase	3.34 (3.07–3.63)	<0.001	2.89 (2.64–3.17)	<0.001	2.89 (2.63–3.17)	<0.001
	Q1	Reference	-	Reference	-	Reference	-
	Q2	3.01 (2.51–3.63)	<0.001	2.51 (2.03–3.11)	<0.001	2.51 (2.04–3.11)	<0.001
	Q3	6.69 (5.63–7.96)	<0.001	4.93 (3.99–6.10)	<0.001	4.93 (3.98–6.10)	<0.001
	Q4	16.95 (14.15–20.31)	<0.001	11.11 (8.96–13.76)	<0.001	11.02 (8.86–13.71)	<0.001
	*p* for trend	<0.001		<0.001		<0.001	

Model 1: Crude (unadjusted). Model 2: Adjusted for age, sex, race, PIR, marital status, education levels. Model 3: Model 2 + adjustments for tobacco and alcohol use.

Currently, HOMA-IR, a widely used surrogate index of IR surrogates, showed a consistent increase in odds for per-SD increase across the three models as follows: Model 1, OR = 5.81, 95% CI: 4.71–7.16; Model 2, OR = 6.85, 95% CI: 5.55–8.45; and Model 3, OR = 6.83, 95% CI: 5.53–8.44. However, notable increases in risk associated with METS-IR, TyG, and TyG’s derivative indexes, which included plasma glucose calculations, were observed across all three models within the intervals from Q3 to Q4. Furthermore, LAP and TyG-ABSI exhibited distinct attenuation patterns with stepwise adjustments. However, this trend pattern did not extend to other indicators.

### 3.3 Nonlinear Associations and Threshold Effects of Different IR Surrogates With CMM

**Fig. 2.**
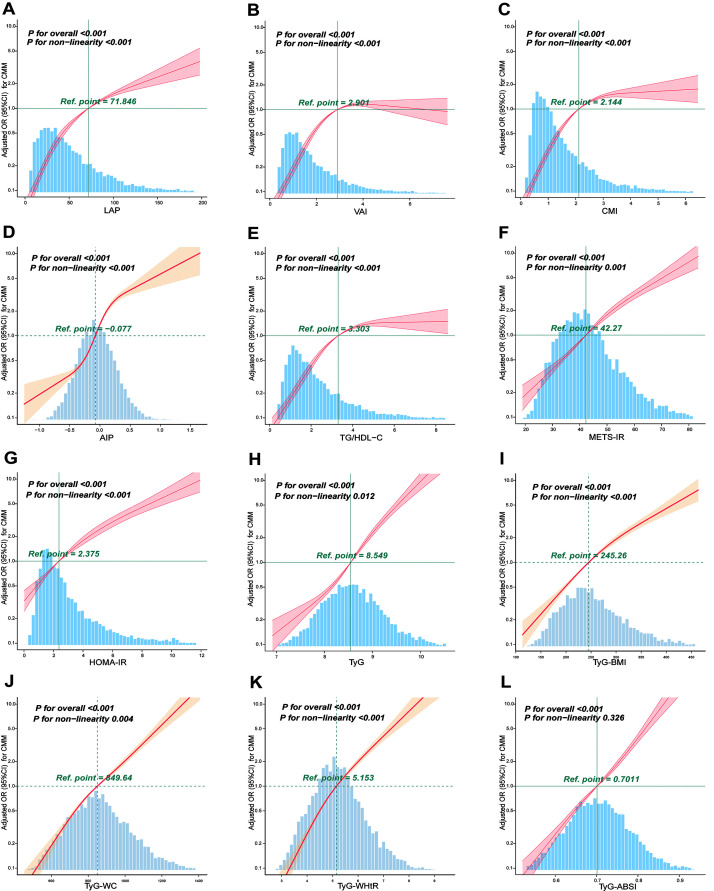
**Multivariable-adjusted RCSs depicting the association between diverse IR surrogates and CMM**. (A) LAP; (B) VAI; (C) CMI; (D) AIP; (E) TG/HDL-C; (F) METS-IR; (G) HOMA-IR; (H) TyG; (I) TyG-BMI; (J) TyG-WC; (K) TyG-WHtR; (L) TyG-ABSI. The models controlled for age, sex, race, PIR, marital status, educational levels, tobacco and alcohol use.

Fig. 2 illustrates the outcomes of RCS analyses after multivariate adjustment, which were utilized to depict the dose-response associations linking various IR surrogates to CMM within this cohort. Among the 12 biomarkers assessed, 11 showed significant nonlinear associations with CMM risk; only TyG-ABSI exhibited a nearly linear relationship (*p* = 0.326).

Analysis of the RCS curves revealed significant changes in those of LAP, VAI, CMI, and TG/HDL-C. Therefore, threshold effect analysis was performed for these variables. The results showed a significant positive correlation between VAI (OR = 2.887, 95% CI: 2.654–3.141) and TG/HDL-C (OR = 2.337, 95% CI: 2.163–2.524) and CMM risk before the inflection point. However, beyond this point, no significant association was found, as detailed in Table 4.

**Table 4.** **Threshold effect analysis of LAP, VAI, CMI, and TG/HDL-C**.

IR surrogates		Adjusted OR (95%CI)	*p*-value
LAP			
	<71.846	1.038 (1.034–1.041)	<0.001
	≥71.846	1.008 (1.005–1.010)	<0.001
	Likelihood ratio test		<0.001
VAI			
	<2.901	2.887 (2.654–3.141)	<0.001
	≥2.901	0.981 (0.884–1.088)	0.718
	Likelihood ratio test		<0.001
CMI			
	<2.144	3.767 (3.356–4.228)	<0.001
	≥2.144	1.081 (1.018–1.147)	0.011
	Likelihood ratio test		<0.001
TG/HDL-C			
	<3.303	2.337 (2.163–2.524)	<0.001
	≥3.303	1.057 (0.978–1.142)	0.161
	Likelihood ratio test		<0.001

Adjusted for age, sex, race, PIR, marital status, education levels, tobacco use and alcohol use.

### 3.4 Subgroup and Sensitivity Analysis

**Fig. 3.**
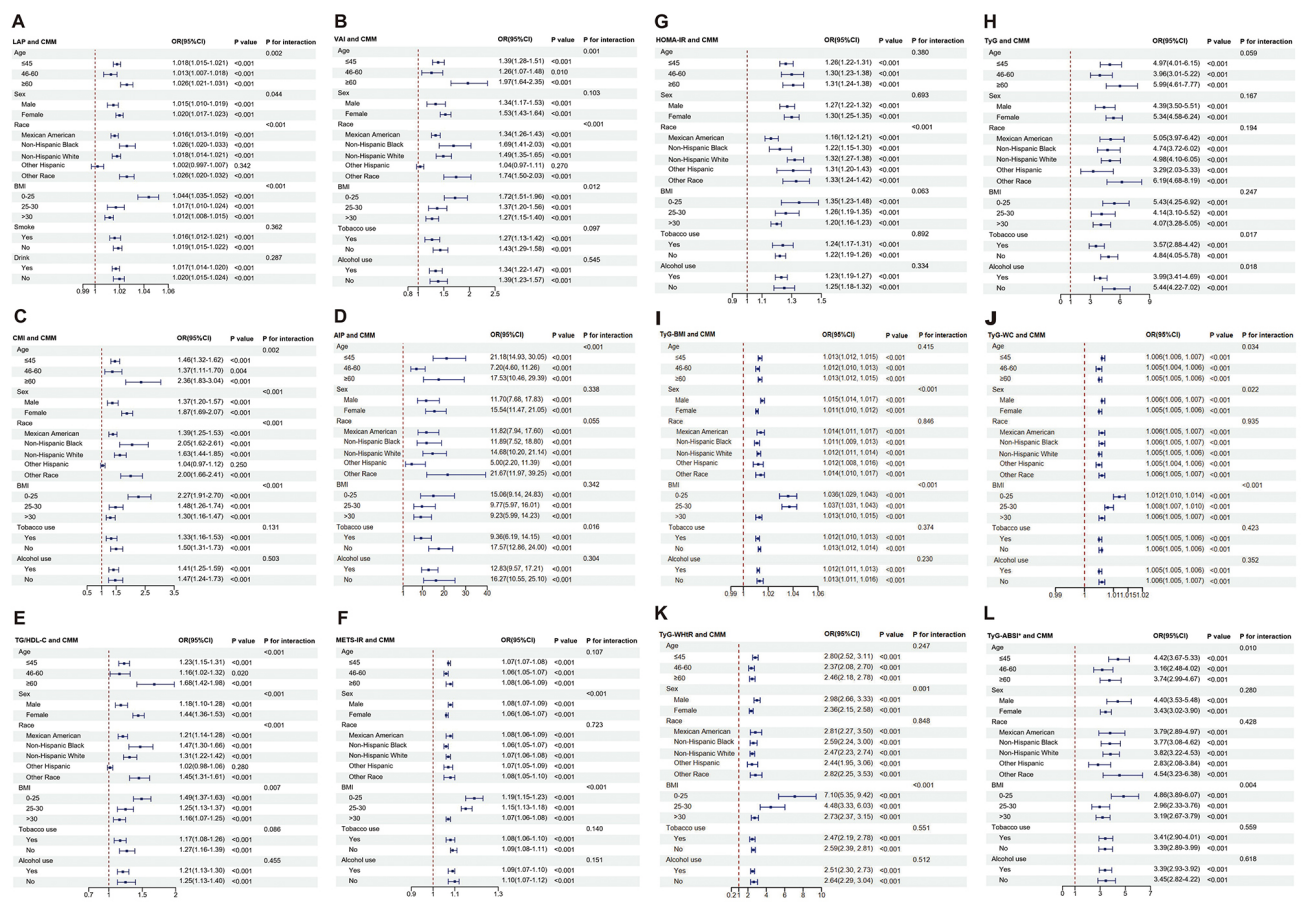
**Stratified analyses of the associations between various surrogate IR indexes and the risk of CMM across different subgroups**. (A–F) Subgroup analyses evaluating the associations of diverse IR surrogates (LAP, VAI, CMI, AIP, TG/HDL-C, and METS-IR) with CMM. (G–L) Subgroup analyses evaluating the associations of IR surrogates (HOMA-IR, TyG, TyG-BMI, TyG-WC, TyG-WHtR, and TyG-ABSI) with CMM. TyG-ABSI values were analyzed after linear transformation (×10), this transformation preserves variable distribution and association directionality while amplifying effect sizes to enhance interpretability and model stability.

Subsequently, we conducted weighted interaction tests and subgroup analyses to assess the stability of the associations between various IR indicators and CMM varied across distinct population subgroups (Fig. 3). The results indicated that in most stratified subgroups, the majority of IR indicators remained significantly and positively associated with CMM. One notable exception was observed in the “Other Hispanic” subgroup, where LAP, VAI, TG/HDL-C, and CMI did not show statistically significant associations with CMM. This may be attributed to the relatively small sample size (n = 866) in this subgroup, which likely resulted in insufficient statistical power.

Interaction tests indicated that age, ethnicity, and BMI showed significant interactions with LAP, VAI, CMI, and TG/HDL-C (*p* for interaction < 0.05). Different indicators also demonstrated varying sensitivities to lifestyle factors. TyG showed significant interactions with smoking and drinking behaviors, whereas TyG-BMI, TyG-WHtR, TyG-WC and TyG-ABSI were not significantly modified by these factors. Notably, HOMA-IR exhibited a distinct pattern: its association with CMM was significantly modified only by race, with no significant influence from other demographic or behavioral variables.

### 3.5 Diagnostic Performance of Different IR Surrogates

**Fig. 4.**
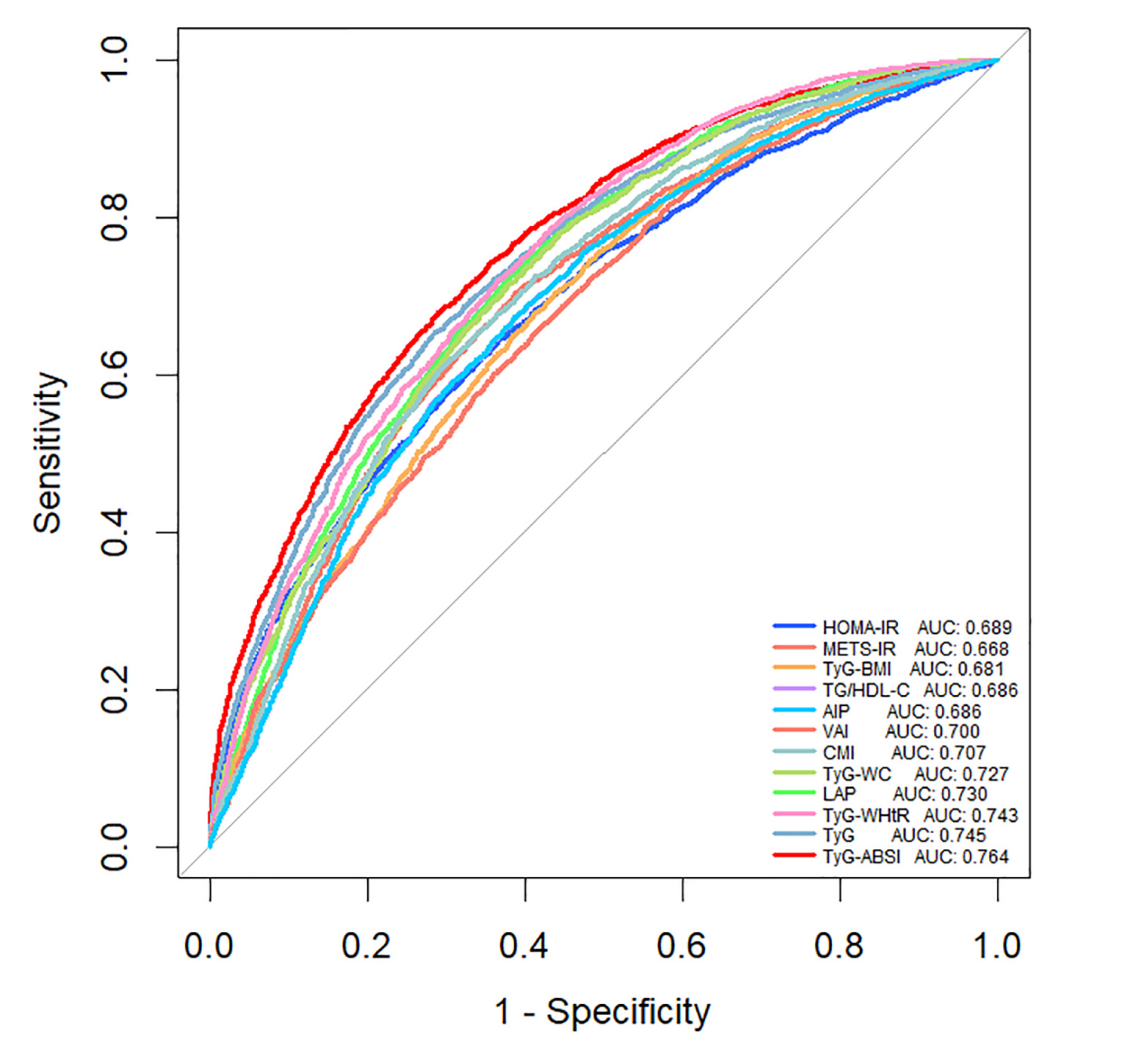
**ROC curves assessing performance of diverse surrogate IR Indexes for CMM**.

To evaluate the capacity of various IR surrogate markers to identify CMM, we performed ROC analyses (**Supplementary Table 2**; Fig. 4). The AUC values ranged from 0.668 (METS-IR) to 0.764 (TyG-ABSI), with TyG-ABSI demonstrating the strongest discriminative ability. Other TyG-related indexes, including TyG-WHtR and TyG, also exhibited strong diagnostic performance. When compared to the widely used surrogate index for IR (HOMA-IR), most indexes showed statistically significant differences in AUC values, except for TyG-BMI, VAI and TG/HDL-C. Notably, TyG-ABSI significantly outperformed HOMA-IR (ΔAUC = 0.075, *p *< 0.001). Visualization of ROC curves further confirmed the conclusion.

We conducted several sensitivity analyses to verify the robustness of our principal findings. Firstly, when we redefined CMM as the presence of ≥3 CMDs, the results remained consistent with our original analysis (**Supplementary Table 3**). Secondly, applying a more restrictive CMM definition (≥2 of: CAD, DM, or stroke) yielded similarly robust associations (**Supplementary Table 4**). Thirdly, further excluding participants with DM (N = 7535) or with hyperlipidemia (N = 3454), the trends remained consistent, which further supports the robustness of our findings (**Supplementary Tables 5,6**). Similarly, after additional adjustments for BMI and waist circumference, the associations of the surrogate IR indexes with CMM remained materially robust (**Supplementary Table 7**). Furthermore, we performed ROC analyses to evaluate model performance based on the sensitivity analyses, consistently demonstrating the superior discriminative ability of TyG-ABSI (**Supplementary Fig. 1**).

## 4. Discussion

To our knowledge, this is the first systematic examination of the association between various IR surrogates and CMM risk using a large, nationally representative sample from NHANES. In the American population, our primary findings indicate that all examined IR surrogate indexes are significantly associated with increased CMM risk. Notably, composite markers that integrate triglycerides, glucose, and anthropometric measures, particularly the TyG-ABSI, exhibited superior discriminative power compared to widely accepted measures like HOMA-IR.

Our analysis confirmed the distinct demographic and socioeconomic profiles among individuals with CMM. Compared to non-CMM participants, those with the condition were significantly older, more likely to be female, reported lower educational attainment, experienced higher poverty rates, and included a greater proportion of widowed individuals. These characteristics are consistent with previously established distributions of risk factors for CMDs [12,19,20]. Furthermore, all assessed IR surrogates were significantly elevated in the CMM group, further emphasizing the important role of IR in the pathogenesis of CMM. We observed a consistent dose-relationship in our analysis; each quartile increment in these indexes was linked to a progressive increase in CMM risk, supporting the idea that IR indicators serve as a continuous risk factor. After adjusting for sociodemographic variables in Model 2, we noted significant increases in the ORs for several indexes, including CMI, AIP and TG/HDL-C, compared to Model 1. To investigate the underlying factors, we incorporated findings from the main subgroup analyses and conducted additional interaction tests involving education, marital status, and PIR (**Supplementary Table 8**). The results indicated that most socioeconomic factors did not exhibit significant interaction effects across various subgroups. In contrast, demographic characteristics, specifically age, sex, and race, appeared to be the primary sources of confounding. Several underlying mechanisms may explain this pattern. First, aging is a well-established risk factor for both aggravated insulin resistance and the development of CMM, thereby acting as a strong confounder [21]. Additionally, significant differences in lipid profiles, particularly HDL-C levels, across sex and racial groups may also drive heterogeneity in risk associations. For example, women typically have higher baseline HDL-C levels than men [22]. These factors may explain why risk estimates for certain indexes changed after adjustment in Model 2. Importantly, the magnitude of the risk varied across the different indexes. For instance, aside from TyG and its derivatives, we observed a notable increase in CMM risk for the LAP when moving from Q3 to Q4. This suggests that high WC and elevated TG levels may exponentially contribute to CMM risk at higher levels. In contrast, although HOMA-IR, often considered a reference surrogate, showed a strong association, no further significant risk increase was observed between the Q4 and Q3 quartiles. This suggests a potential plateau or saturation effect in HOMA-IR’s ability to distinguish between different levels of IR at the higher end of the spectrum. Strikingly, we observed high ORs in the highest quartile for TyG-derived indexes that incorporate fasting glucose. This implies that considering both glycemic status and adiposity metrics (notably BMI) alongside triglyceride levels may more effectively capture the extent of metabolic dysregulation driving CMM. The robust performance of the TyG and its derivative indexes highlights their utility as simple, insulin-free markers in both clinical and epidemiological research, owing to their ease of calculation [9,14,23].

Furthermore, RCS analyses uncovered non-linear relationships between most IR surrogates and CMM prevalence. This non-linearity, exemplified by the threshold effects observed for VAI and TG/HDL-C, carries significant clinical implications. It suggests that for certain indexes, a critical range exists where risk accelerates sharply, beyond which the marginal contribution to risk diminishes, or other factors potentially become more dominant drivers of CMM. Conversely, the near-linear relationship for TyG-ABSI implies that increases in this indexes correspond to a relatively consistent rise in CMM risk across the observed spectrum. This linearity makes them easier to construct risk prediction models and interpret clinically. The nonlinear relationships and threshold effects revealed by RCS analysis suggest that in the future, it may be necessary to set specific risk thresholds for different IR indicators, rather than a single linear assumption.

Our analysis demonstrates that low-cost IR surrogates, especially TyG-derived indexes such as TyG-ABSI and TyG-WHtR, are highly effective in identifying high-risk CMM populations, even surpassing HOMA-IR. Notably, TyG-ABSI achieved the highest discriminative accuracy among all markers evaluated, as reflected by its superior AUC value. The enhanced performance of TyG-ABSI can be largely attributed to its integration of the ABSI. Unlike conventional measures such as BMI, ABSI is specifically designed to capture body shape and central obesity by normalizing waist circumference for height and BMI. This provides a more accurate representation of abdominal fat distribution, independent of overall body weight [24]. When combined with the TyG index, TyG-ABSI synergistically reflects both metabolic dysfunction and central adiposity, allowing for more comprehensive cardiometabolic risk phenotyping. Although TyG-ABSI requires more complex calculation than TyG or TyG-BMI, it relies only on routinely collected anthropometric and biochemical parameters. This does not hinder its clinical implementation in the era of accessible digital tools. The capacity of TyG-ABSI to enhance the differentiation of cardiometabolic status using routine clinical data supports its utility as a practical and efficient tool for large-scale screening and routine clinical assessment. Consequently, incorporating TyG-ABSI into standard evaluations may refine the detection of high-burden individuals and provide valuable insights for cardiometabolic health assessment.

### Strengths and Limitations

This study has several key strengths. First, the use of a nationally representative sample from NHANES enhances the generalizability and reliability of our findings. Second, we systematically characterized dose-response relationships and identified critical inflection points between various IR surrogates and CMM risk, providing essential evidence to guide future mechanistic and interventional research. Third, by directly comparing 12 IR surrogates, we established TyG-ABSI as the indicator most strongly associated with CMM in this population, and its superior discriminative performance was validated through multiple sensitivity analyses. Finally, the integration of stratified analyses and various sensitivity frameworks strengthens the internal validity of our findings. To our knowledge, this is the first study to comprehensively evaluate the associations between 12 IR indexes and CMM risk, as well as the first to demonstrate the discriminative utility of TyG-ABSI for CMM identification, thereby addressing a critical knowledge gap in the field.

Nevertheless, several limitations should be acknowledged. First, the cross-sectional design prevents us from establishing causality or determining temporal sequences. Although previous studies in the United States have confirmed the predictive value of TyG-ABSI for cardiovascular disease mortality, its temporal relationship with CMM incidence and cardiovascular outcomes remains unclear [15]. To address these issues, future research should prioritize longitudinal designs that include long-term follow-ups. Large-scale prospective cohorts, such as the China Health and Retirement Longitudinal Study (CHARLS) or the English Longitudinal Study of Ageing (ELSA), would be ideal for validation. These studies would enable researchers to track baseline TyG-ABSI levels and monitor the incidence of CMM over time, thus confirming the temporal relationship and predictive validity of this novel index. Second, reliance on self-reported diagnoses for CMM components may introduce potential recall bias and misclassification, particularly for conditions like diabetes or cardiovascular diseases. Third, we provide the proportion of missing data for key variables in the full NHANES sample prior to exclusions in **Supplementary Table 9**. It is important to note that although our analysis relied on a complete-case approach to ensure data integrity, the exclusion of participants with missing data or those who did not meet specific inclusion criteria may introduce selection bias, potentially limiting the external validity of our findings. Fourth, the inclusion of diabetes and dyslipidemia in the CMM definition may introduce diagnostic incorporation bias and potentially inflate both the strength of associations and AUC estimates, as it overlaps with several IR indexes. While this overlap is a common feature of the metabolic indexes, it is an important limitation when interpreting our results. Fifth, all participants in this study were recruited from the United States, which means that ethnic variations in body composition and metabolic phenotypes could significantly affect the performance and generalizability of IR surrogates in other populations. Emerging evidence from Chinese cohorts suggests that there are significant associations between TyG-ABSI and conditions such as stroke and Cardiovascular-Kidney-Metabolic (CKM) syndrome. However, the specific prognostic utility of TyG-ABSI specifically for CMM in non-U.S. populations remains unestablished [25,26]. Consequently, further investigations are needed to determine the generalizability of our findings across diverse ethnic groups. Sixth, although HOMA-IR was pre-specified as the reference standard without performing undirected pairwise comparisons among all indicators, and the DeLong test showed *p* < 0.001 between TyG-ABSI and sub-optimal metrics, suggesting that multiplicity issues have limited impact on the primary findings, it should be stated that these results remain exploratory in nature. Furthermore, the ROC/AUC estimation and comparison based on the DeLong method did not incorporate the complex survey design elements of NHANES (such as weighting, stratification, and clustering). While this approach helps ensure statistical stability, the results should be interpreted as reflecting cross-sectional discriminative ability within the sample rather than nationally representative predictive estimates. Lastly, even though we accounted for a comprehensive range of confounding variables, we cannot rule out residual bias from the unmeasured factors such as dietary patterns, genetic predisposition, or medication adherence [27,28,29].

## 5. Conclusions

This study systematically evaluates 12 IR surrogates and identifies the TyG-ABSI as a clinically practical and economically viable biomarker for stratifying CMM risk. By simultaneously capturing visceral adiposity and metabolic dysregulation, TyG-ABSI addresses key limitations in current screening tools, offering a transformative approach to early detection of CMM and tailored risk management. These findings have significant implications for redefining preventive strategies in high-risk populations.

## Data Availability

The data were obtained from publicly available sources, as previously stated.

## Abbreviations

CMM, cardiometabolic multimorbidity; CMDs, cardiometabolic diseases; IR, insulin resistance; MACE, major adverse cardiovascular events; LAP, lipid accumulation product; VAI, visceral adiposity index; CMI, cardiometabolic index; AIP, atherogenic index of plasma; TG/HDL-C, triglyceride to high-density lipoprotein cholesterol ratio; METS-IR, metabolic score for insulin resistance; HOMA-IR, homeostatic model assessment of insulin resistance; TyG, triglyceride-glucose; TyG-BMI, TyG-body mass index; TyG-WC, TyG-waist circumference; TyG-WHtR, TyG-waist-to-height ratio; TyG-ABSI, TyG-a body shape index; NHANES, National Health and Nutrition Examination Survey; WC, waist circumference; TG, triglyceride; TC, total cholesterol; LDL-C, low-density lipoprotein cholesterol; HDL-C, high-density lipoprotein cholesterol; BMI, body mass index; FPG, fasting plasma glucose; OGTT 2hPG, 2-hour oral glucose tolerance test; PIR, poverty income ratio; HTN, hypertension; CAD, coronary artery disease; DM, diabetes mellitus; OR, odds ratios; CI, confidence interval; SD, standard deviation; RCS, restricted cubic splines; ROC, receiver operating characteristic; AUC, area under the curve; ASCVD, atherosclerotic cardiovascular disease; CDC, Centers for Disease Control and Prevention; SBP, systolic blood pressure; DBP, diastolic blood pressure; NCEP-ATP III, the National Cholesterol Education Program Adult Treatment Panel III; ANOVA, analysis of variance.
