# Supplementary material for: Comparative Analysis of Surrogate Insulin Resistance Indexes in Cardiometabolic Multimorbidity: A Cross-Sectional Study
Source: Rev Cardiovasc Med. 2026 Jun 23;27(6):46005. doi: 10.31083/RCM46005 (PMC13339193; doi:10.31083/RCM46005)
Supplement: Supplementary file 1 [file 2153-8174-27-6-46005-s1.zip › Supplementary Material.docx]

**Supplementary Table 1**. **Operational Definitions for CMM Components based on NHANES Database.**

| Components | Detailed Explanation |
| --- | --- |
| Hypertension | 1) The final compared SBP and DBP values are derived from the average of multiple blood pressure measurements;  2) A self-reported diagnosis of hypertension was identified by a "Yes" response to the question: "Have you ever been told by a doctor or other health professional that you had hypertension, also called high blood pressure?" (BPQ020);  3) Current use of antihypertensive agents (BPQ050A=1). |
| Diabetes | 1) The cutoff values for biochemical indicators in the diagnostic criteria for diabetes are based on recommendations from the American Diabetes Association (ADA);  2) Self‑reported diabetes was defined as a “Yes” response to: “Have you ever been told by a doctor or other health professional that you had diabetes?” (DIQ010);  3) Currently using insulin (DIQ050=1) or oral hypoglycemic medications (DIQ070=1). |
| Hyperlipidemia | 1) The diagnostic cutoff values for blood lipid indicators in hyperlipidemia are based on the criteria outlined in the NCEP-ATP III guidelines;  2) Current use of lipid-lowering medications was confirmed through the identification of relevant drug codes (RXDRSC1). |
| CAD | Self‑reported CAD was defined as a “Yes” to any of the following: “Has a doctor or other health professional ever told you that you had coronary heart disease? (MCQ160C=1), “… had angina pectoris?” (MCQ160D=1), or “… had a heart attack (myocardial infarction)?” (MCQ160E=1). |
| Stroke | Stroke was defined as a “Yes” response to: “Has a doctor or other health professional ever told you that you had a stroke?” (MCQ160F=1). |

**Supplementary Table 2**. **ROC test: evaluating the utility of various IR indicators in identifying CMM.**

| Test | AUC | 95%CI | Cutoff value | Specificity | Sensitivity | *p* Value | *p* Value# |
| --- | --- | --- | --- | --- | --- | --- | --- |
| HOMA-IR | 0.689 | 0.679-0.700 | 2.5319 | 0.6307 | 0.6448 | <0.001 | - |
| LAP | 0.730 | 0.720-0.740 | 39.6546 | 0.6164 | 0.7253 | <0.001 | <0.001 |
| VAI | 0.700 | 0.690-0.711 | 1.3677 | 0.6127 | 0.7016 | <0.001 | 0.059 |
| CMI | 0.707 | 0.697-0.717 | 1.3291 | 0.7018 | 0.6115 | <0.001 | 0.001 |
| AIP | 0.686 | 0.675-0.696 | -0.0893 | 0.6127 | 0.6726 | <0.001 | <0.001 |
| TG/HDL-C | 0.686 | 0.675-0.696 | 1.8652 | 0.6127 | 0.6724 | <0.001 | 0.525 |
| METS-IR | 0.668 | 0.657-0.679 | 41.6942 | 0.5840 | 0.6557 | <0.001 | <0.001 |
| TyG | 0.745 | 0.735-0.755 | 8.6397 | 0.7134 | 0.6530 | <0.001 | <0.001 |
| TyG-BMI | 0.681 | 0.671-0.692 | 241.416 | 0.5887 | 0.6760 | <0.001 | 0.094 |
| TyG-WC | 0.727 | 0.717-0.737 | 836.5432 | 0.6190 | 0.7159 | <0.001 | <0.001 |
| TyG-WHtR | 0.743 | 0.734-0.753 | 5.0883 | 0.6334 | 0.7198 | <0.001 | <0.001 |
| TyG-ABSI | 0.764 | 0.755-0.774 | 0.7081 | 0.7093 | 0.6790 | <0.001 | <0.001 |

Note: *p* value#: comparison of HOMA-IR and other surrogate IR indexes.

**Supplementary Table 3**. **Sensitivity analysis of different surrogate IR indexes on the risk of ≥3 CMDs in study population.**

|  | Model 1 | | Model 2 | | Model 3 | |
| --- | --- | --- | --- | --- | --- | --- |
|  | OR (95% CI) | *p* Value | OR (95% CI) | *p* Value | OR (95% CI) | *p* Value |
| LAP |  |  |  |  |  |  |
| Per-SD increase | 2.05(1.75-2.40) | <0.001 | 2.24(1.87-2.68) | <0.001 | 2.21(1.85-2.66) | <0.001 |
| Q1 | Reference | - | Reference | - | Reference | - |
| Q2 | 2.97(2.05-4.29) | <0.001 | 2.06(1.43-2.98) | <0.001 | 2.08(1.44-3.02) | <0.001 |
| Q3 | 8.83(6.04-12.93) | <0.001 | 6.29(4.28-9.25) | <0.001 | 6.28(4.26-9.27) | <0.001 |
| Q4 | 18.60(13.05-26.50) | <0.001 | 16.02(11.09-23.14) | <0.001 | 15.77(10.84-22.94) | <0.001 |
| *P* for trend | <0.001 |  | <0.001 |  | <0.001 |  |
| VAI |  |  |  |  |  |  |
| Per-SD increase | 1.75(1.50-2.04) | <0.001 | 1.82(1.52-2.18) | <0.001 | 1.80(1.50-2.15) | <0.001 |
| Q1 | Reference | - | Reference | - | Reference | - |
| Q2 | 2.69(2.04-3.55) | <0.001 | 2.37(1.80-3.13) | <0.001 | 2.37(1.79-3.14) | <0.001 |
| Q3 | 6.15(4.86-7.77) | <0.001 | 5.39(4.18-6.95) | <0.001 | 5.38(4.17-6.93) | <0.001 |
| Q4 | 14.93(11.69-19.06) | <0.001 | 15.96(12.43-20.48) | <0.001 | 15.61(12.11-20.12) | <0.001 |
| *P* for trend | <0.001 |  | <0.001 |  | <0.001 |  |
| CMI |  |  |  |  |  |  |
| Per-SD increase | 1.77(1.50-2.08) | <0.001 | 1.94(1.60-2.35) | <0.001 | 1.91(1.57-2.33) | <0.001 |
| Q1 | Reference | - | Reference | - | Reference | - |
| Q2 | 3.09(2.29-4.17) | <0.001 | 2.48(1.80-3.43) | <0.001 | 2.49(1.80-3.44) | <0.001 |
| Q3 | 7.56(5.70-10.02) | <0.001 | 6.42(4.82-8.56) | <0.001 | 6.37(4.77-8.50) | <0.001 |
| Q4 | 17.69(13.65-22.92) | <0.001 | 19.93(15.06-26.37) | <0.001 | 19.46(14.67-25.81) | <0.001 |
| *P* for trend | <0.001 |  | <0.001 |  | <0.001 |  |
| AIP |  |  |  |  |  |  |
| Per-SD increase | 2.24(2.10-2.39) | <0.001 | 2.64(2.43-2.87) | <0.001 | 2.61(2.39-2.84) | <0.001 |
| Q1 | Reference | - | Reference | - | Reference | - |
| Q2 | 2.90(2.06-4.08) | <0.001 | 2.67(1.87-3.80) | <0.001 | 2.69(1.88-3.84) | <0.001 |
| Q3 | 5.65(4.34-7.37) | <0.001 | 5.10(3.88-6.71) | <0.001 | 5.06(3.85-6.65) | <0.001 |
| Q4 | 13.07(10.16-16.81) | <0.001 | 16.01(12.13-21.12) | <0.001 | 15.67(11.87-20.69) | <0.001 |
| *P* for trend | <0.001 |  | <0.001 |  | <0.001 |  |
| TG/HDL-C |  |  |  |  |  |  |
| Per-SD increase | 1.60(1.38-1.85) | <0.001 | 1.73(1.46-2.05) | <0.001 | 1.71(1.44-2.03) | <0.001 |
| Q1 | Reference | - | Reference | - | Reference | - |
| Q2 | 2.87(2.04-4.04) | <0.001 | 2.62(1.85-3.73) | <0.001 | 2.65(1.86-3.77) | <0.001 |
| Q3 | 5.64(4.33-7.34) | <0.001 | 5.08(3.86-6.67) | <0.001 | 5.04(3.84-6.62) | <0.001 |
| Q4 | 13.08(10.18-16.81) | <0.001 | 15.94(12.10-21.01) | <0.001 | 15.62(11.84-20.60) | <0.001 |
| *P* for trend | <0.001 |  | <0.001 |  | <0.001 |  |
| METS-IR |  |  |  |  |  |  |
| Per-SD increase | 2.00(1.86-2.14) | <0.001 | 2.68(2.45-2.92) | <0.001 | 2.68(2.45-2.94) | <0.001 |
| Q1 | Reference | - | Reference | - | Reference | - |
| Q2 | 3.33(2.39-4.64) | <0.001 | 2.60(1.86-3.63) | <0.001 | 2.60(1.85-3.64) | <0.001 |
| Q3 | 5.16(3.70-7.19) | <0.001 | 5.01(3.62-6.94) | <0.001 | 5.06(3.65-7.04) | <0.001 |
| Q4 | 10.79(7.89-14.76) | <0.001 | 15.41(11.34-20.94) | <0.001 | 15.55(11.42-21.18) | <0.001 |
| *P* for trend | <0.001 |  | <0.001 |  | <0.001 |  |
| HOMA-IR |  |  |  |  |  |  |
| Per-SD increase | 2.05(1.66-2.53) | <0.001 | 2.19(1.72-2.77) | <0.001 | 2.19(1.73-2.78) | <0.001 |
| Q1 | Reference | - | Reference | - | Reference | - |
| Q2 | 2.04(1.50-2.76) | <0.001 | 1.76(1.32-2.35) | <0.001 | 1.76(1.32-2.36) | <0.001 |
| Q3 | 3.74(2.74-5.09) | <0.001 | 3.35(2.44-4.58) | <0.001 | 3.34(2.45-4.57) | <0.001 |
| Q4 | 11.1(8.54-14.42) | <0.001 | 12.07(9.45-15.41) | <0.001 | 12.17(9.50-15.57) | <0.001 |
| *P* for trend | <0.001 |  | <0.001 |  | <0.001 |  |
| TyG |  |  |  |  |  |  |
| Per-SD increase | 3.11(2.84-3.41) | <0.001 | 3.39(3.03-3.78) | <0.001 | 3.37(3.01-3.77) | <0.001 |
| Q1 | Reference | - | Reference | - | Reference | - |
| Q2 | 3.30(2.43-4.48) | <0.001 | 2.39(1.77-3.23) | <0.001 | 2.41(1.78-3.27) | <0.001 |
| Q3 | 7.37(5.56-9.76) | <0.001 | 5.34(3.98-7.17) | <0.001 | 5.32(3.97-7.14) | <0.001 |
| Q4 | 26.83(20.46-35.19) | <0.001 | 23.06(17.20-30.91) | <0.001 | 22.81(17.02-30.57) | <0.001 |
| *P* for trend | <0.001 |  | <0.001 |  | <0.001 |  |
| TyG-BMI |  |  |  |  |  |  |
| Per-SD increase | 2.00(1.87-2.14) | <0.001 | 2.54(2.33-2.77) | <0.001 | 2.55(2.33-2.79) | <0.001 |
| Q1 | Reference | - | Reference | - | Reference | - |
| Q2 | 3.72(2.69-5.14) | <0.001 | 2.67(1.94-3.69) | <0.001 | 2.70(1.95-3.75) | <0.001 |
| Q3 | 5.45(3.82-7.76) | <0.001 | 4.64(3.28-6.57) | <0.001 | 4.69(3.29-6.68) | <0.001 |
| Q4 | 11.49(8.33-15.85) | <0.001 | 13.65(9.96-18.72) | <0.001 | 13.76(9.95-19.01) | <0.001 |
| *P* for trend | <0.001 |  | <0.001 |  | <0.001 |  |
| TyG-WC |  |  |  |  |  |  |
| Per-SD increase | 2.71(2.50-2.94) | <0.001 | 3.32(3.02-3.64) | <0.001 | 3.31(3.00-3.65) | <0.001 |
| Q1 | Reference | - | Reference | - | Reference | - |
| Q2 | 3.31(2.24-4.90) | <0.001 | 2.29(1.55-3.37) | <0.001 | 2.27(1.53-3.37) | <0.001 |
| Q3 | 8.40(5.62-12.55) | <0.001 | 6.01(4.04-8.96) | <0.001 | 6.05(4.06-9.02) | <0.001 |
| Q4 | 20.46(14.04-29.82) | <0.001 | 18.82(12.95-27.36) | <0.001 | 18.56(12.70-27.13) | <0.001 |
| *P* for trend | <0.001 |  | <0.001 |  | <0.001 |  |
| TyG-WHtR |  |  |  |  |  |  |
| Per-SD increase | 2.91(2.68-3.17) | <0.001 | 3.26(2.96-3.59) | <0.001 | 3.26(2.95-3.60) | <0.001 |
| Q1 | Reference | - | Reference | - | Reference | - |
| Q2 | 4.41(2.94-6.62) | <0.001 | 2.74(1.82-4.15) | <0.001 | 2.78(1.83-4.22) | <0.001 |
| Q3 | 10.44(6.65-16.37) | <0.001 | 6.44(4.07-10.19) | <0.001 | 6.50(4.11-10.30) | <0.001 |
| Q4 | 28.92(19.04-43.95) | <0.001 | 20.63(13.51-31.52) | <0.001 | 20.68(13.51-31.64) | <0.001 |
| *P* for trend | <0.001 |  | <0.001 |  | <0.001 |  |
| TyG-ABSI |  |  |  |  |  |  |
| Per-SD increase | 3.98(3.59-4.41) | <0.001 | 3.62(3.22-4.07) | <0.001 | 3.58(3.17-4.03) | <0.001 |
| Q1 | Reference | - | Reference | - | Reference | - |
| Q2 | 4.06(2.73-6.06) | <0.001 | 3.17(2.09-4.80) | <0.001 | 3.13(2.07-4.72) | <0.001 |
| Q3 | 9.87(6.65-14.67) | <0.001 | 6.69(4.40-10.18) | <0.001 | 6.59(4.34-10.00) | <0.001 |
| Q4 | 39.12(26.28-58.21) | <0.001 | 24.59(15.64-38.65) | <0.001 | 23.68(15.04-37.28) | <0.001 |
| *P* for trend | <0.001 |  | <0.001 |  | <0.001 |  |

Model 1: Crude (unadjusted).

Model 2: Adjusted for age, sex, race, PIR, marital status, education levels.

Model 3: Model 2 + adjustments for tobacco and alcohol use.

**Supplementary Table 4**. **Sensitivity analysis of different surrogate IR indexes on the risk of CMM (≥2 of: CAD, DM, or stroke).**

|  | Model 1 | | Model 2 | | Model 3 | |
| --- | --- | --- | --- | --- | --- | --- |
|  | OR (95% CI) | *p* Value | OR (95% CI) | *p* Value | OR (95% CI) | *p* Value |
| LAP |  |  |  |  |  |  |
| Per-SD increase | 1.24(1.10-1.41) | <0.001 | 1.24(1.08-1.41) | 0.002 | 1.19(1.07-1.31) | <0.001 |
| Q1 | Reference | - | Reference | - | Reference | - |
| Q2 | 2.63(1.48-4.68) | 0.001 | 1.75(0.96-3.20) | 0.066 | 1.72(0.92-3.22) | 0.09 |
| Q3 | 4.80(2.69-8.58) | <0.001 | 3.13(1.71-5.73) | <0.001 | 2.98(1.59-5.59) | <0.001 |
| Q4 | 7.66(4.35-13.50) | <0.001 | 5.51(3.05-9.97) | <0.001 | 5.06(2.79-9.16) | <0.001 |
| *P* for trend | <0.001 |  | <0.001 |  | <0.001 |  |
| VAI |  |  |  |  |  |  |
| Per-SD increase | 1.20(1.08-1.32) | <0.001 | 1.19(1.09-1.31) | <0.001 | 1.18(1.10-1.26) | <0.001 |
| Q1 | Reference | - | Reference | - | Reference | - |
| Q2 | 2.19(1.56-3.09) | <0.001 | 1.90(1.34-2.68) | <0.001 | 1.84(1.28-2.64) | 0.001 |
| Q3 | 3.54(2.59-4.85) | <0.001 | 2.96(2.09-4.18) | <0.001 | 3.13(2.14-4.58) | <0.001 |
| Q4 | 5.46(3.79-7.86) | <0.001 | 4.88(3.22-7.37) | <0.001 | 5.76(3.65-9.08) | <0.001 |
| *P* for trend | <0.001 |  | <0.001 |  | <0.001 |  |
| CMI |  |  |  |  |  |  |
| Per-SD increase | 1.21(1.10-1.32) | <0.001 | 1.20(1.10-1.31) | <0.001 | 1.18(1.11-1.26) | <0.001 |
| Q1 | Reference | - | Reference | - | Reference | - |
| Q2 | 3.22(2.22-4.97) | <0.001 | 2.52(1.65-3.86) | <0.001 | 2.44(1.56-3.81) | <0.001 |
| Q3 | 4.49(3.00-6.72) | <0.001 | 3.25(2.20-4.80) | <0.001 | 3.21(2.13-4.82) | <0.001 |
| Q4 | 7.79(5.21-11.65) | <0.001 | 6.17(4.07-9.35) | <0.001 | 6.59(4.16-10.44) | <0.001 |
| *P* for trend | <0.001 |  | <0.001 |  | <0.001 |  |
| AIP |  |  |  |  |  |  |
| Per-SD increase | 1.68(1.51-1.87) | <0.001 | 1.68(1.49-1.90) | <0.001 | 1.66(1.47-1.87) | <0.001 |
| Q1 | Reference | - | Reference | - | Reference | - |
| Q2 | 2.46(1.68-3.59) | <0.001 | 2.13(1.43-3.18) | <0.001 | 2.16(1.45-3.22) | <0.001 |
| Q3 | 3.18(2.18-4.64) | <0.001 | 2.41(1.66-3.47) | <0.001 | 2.35(1.63-3.40) | <0.001 |
| Q4 | 5.66(3.91-8.20) | <0.001 | 4.87(3.32-7.16) | <0.001 | 4.74(3.23-6.95) | <0.001 |
| *P* for trend | <0.001 |  | <0.001 |  | <0.001 |  |
| TG/HDL-C |  |  |  |  |  |  |
| Per-SD increase | 1.18(1.09-1.28) | <0.001 | 1.18(1.09-1.27) | <0.001 | 1.17(1.10-1.24) | <0.001 |
| Q1 | Reference | - | Reference | - | Reference | - |
| Q2 | 2.41(1.64-3.54) | <0.001 | 2.06(1.37-3.08) | <0.001 | 2.04(1.34-3.10) | <0.001 |
| Q3 | 3.19(2.19-4.65) | <0.001 | 2.39(1.66-3.46) | <0.001 | 2.40(1.63-3.52) | <0.001 |
| Q4 | 5.65(3.90-8.18) | <0.001 | 4.80(3.28-7.02) | <0.001 | 5.36(3.48-8.26) | <0.001 |
| *P* for trend | <0.001 |  | <0.001 |  | <0.001 |  |
| METS-IR |  |  |  |  |  |  |
| Per-SD increase | 1.49(1.36-1.63) | <0.001 | 1.82(1.61-2.06) | <0.001 | 1.65(1.46-1.88) | <0.001 |
| Q1 | Reference | - | Reference | - | Reference | - |
| Q2 | 2.47(1.48-4.14) | <0.001 | 1.69(1.02-2.81) | 0.042 | 1.54(0.92-2.59) | 0.101 |
| Q3 | 3.57(2.18-5.83) | <0.001 | 2.91(1.82-4.66) | <0.001 | 2.56(1.58-4.14) | <0.001 |
| Q4 | 5.05(3.12-8.17) | <0.001 | 5.81(3.65-9.26) | <0.001 | 4.61(2.90-7.32) | <0.001 |
| *P* for trend | <0.001 |  | <0.001 |  | <0.001 |  |
| HOMA-IR |  |  |  |  |  |  |
| Per-SD increase | 1.29(1.14-1.46) | <0.001 | 1.29(1.18-1.42) | <0.001 | 1.26(1.15-1.39) | <0.001 |
| Q1 | Reference | - | Reference | - | Reference | - |
| Q2 | 1.67(1.07-2.59) | 0.024 | 1.39(0.91-2.12) | 0.129 | 1.31(0.84-2.03) | 0.229 |
| Q3 | 3.25(2.23-4.74) | <0.001 | 2.69(1.83-3.96) | <0.001 | 2.38(1.62-3.49) | <0.001 |
| Q4 | 6.38(4.45-9.15) | <0.001 | 5.55(3.93-7.83) | <0.001 | 4.38(3.08-6.24) | <0.001 |
| *P* for trend | <0.001 |  | <0.001 |  | <0.001 |  |
| TyG |  |  |  |  |  |  |
| Per-SD increase | 1.98(1.80-2.19) | <0.001 | 1.90(1.69-2.14) | <0.001 | 1.95(1.73-2.19) | <0.001 |
| Q1 | Reference | - | Reference | - | Reference | - |
| Q2 | 2.49(1.59-3.91) | <0.001 | 1.63(1.05-2.52) | 0.03 | 1.70(1.08-2.67) | 0.023 |
| Q3 | 4.21(2.80-6.35) | <0.001 | 2.54(1.68-3.84) | <0.001 | 2.62(1.66-4.13) | <0.001 |
| Q4 | 10.30(6.96-15.25) | <0.001 | 6.12(4.13-9.33) | <0.001 | 7.05(4.47-11.12) | <0.001 |
| *P* for trend | <0.001 |  | <0.001 |  | <0.001 |  |
| TyG-BMI |  |  |  |  |  |  |
| Per-SD increase | 1.48(1.35-1.61) | <0.001 | 1.76(1.57-1.98) | <0.001 | 1.74(1.53-1.96) | <0.001 |
| Q1 | Reference | - | Reference | - | Reference | - |
| Q2 | 3.32(1.96-5.63) | <0.001 | 2.16(1.28-3.63) | 0.011 | 2.18(1.28-3.70) | 0.011 |
| Q3 | 4.54(2.57-8.00) | <0.001 | 3.48(2.01-6.02) | <0.001 | 3.45(1.97-6.04) | <0.001 |
| Q4 | 6.11(3.60-10.36) | <0.001 | 6.32(3.73-10.71) | <0.001 | 6.22(3.62-10.67) | <0.001 |
| *P* for trend | <0.001 |  | <0.001 |  | <0.001 |  |
| TyG-WC |  |  |  |  |  |  |
| Per-SD increase | 1.91(1.72-2.12) | <0.001 | 2.03(1.78-2.33) | <0.001 | 1.99(1.72-2.29) | <0.001 |
| Q1 | Reference | - | Reference | - | Reference | - |
| Q2 | 2.03(1.15-3.60) | 0.015 | 1.22(0.69-2.14) | 0.489 | 1.21(0.68-2.13) | 0.519 |
| Q3 | 5.34(2.93-9.76) | <0.001 | 3.03(1.67-5.50) | <0.001 | 3.04(1.67-5.53) | <0.001 |
| Q4 | 8.25(4.66-14.61) | <0.001 | 5.34(3.06-9.32) | <0.001 | 5.09(2.88-8.99) | <0.001 |
| *P* for trend | <0.001 |  | <0.001 |  | <0.001 |  |
| TyG-WHtR |  |  |  |  |  |  |
| Per-SD increase | 1.96(1.77-2.16) | <0.001 | 2.04(1.78-2.34) | <0.001 | 2.00(1.74-2.31) | <0.001 |
| Q1 | Reference | - | Reference | - | Reference | - |
| Q2 | 4.01(2.05-7.87) | <0.001 | 2.28(1.14-4.56) | 0.020 | 2.33(1.16-4.67) | 0.018 |
| Q3 | 7.44(3.48-15.90) | <0.001 | 4.09(1.90-8.84) | <0.001 | 4.10(1.89-8.90) | <0.001 |
| Q4 | 13.50(6.67-27.33) | <0.001 | 8.09(3.91-16.72) | <0.001 | 7.92(3.80-16.52) | <0.001 |
| *P* for trend | <0.001 |  | <0.001 |  | <0.001 |  |
| TyG-ABSI |  |  |  |  |  |  |
| Per-SD increase | 2.64(2.35-2.96) | <0.001 | 2.07(1.81-2.37) | <0.001 | 2.04(1.79-2.32) | <0.001 |
| Q1 | Reference | - | Reference | - | Reference | - |
| Q2 | 4.43(2.35-8.32) | <0.001 | 2.67(1.39-5.13) | 0.003 | 2.43(1.26-4.70) | 0.008 |
| Q3 | 11.5(6.51-20.32) | <0.001 | 5.31(2.92-9.65) | <0.001 | 4.47(2.58-8.71) | <0.001 |
| Q4 | 29.2(15.97-53.37) | <0.001 | 10.38(5.45-19.77) | <0.001 | 9.18(4.88-17.24) | <0.001 |
| *P* for trend | <0.001 |  | <0.001 |  | <0.001 |  |

Model 1: Crude (unadjusted).

Model 2: Adjusted for age, sex, race, PIR, marital status, education levels.

Model 3: Model 2 + adjustments for tobacco use, alcohol use, hypertension (HTN) and dyslipidemia.

**Supplementary Table 5**. **Sensitivity analysis of different surrogate IR indexes on the risk of CMM in participants without DM (N=7,535).**

|  | Model 1 | | Model 2 | | Model 3 | |
| --- | --- | --- | --- | --- | --- | --- |
|  | OR (95% CI) | *p* Value | OR (95% CI) | *p* Value | OR (95% CI) | *p* Value |
| LAP |  |  |  |  |  |  |
| Per-SD increase | 2.17(1.83-2.59) | <0.001 | 2.26(1.89-2.70) | <0.001 | 2.25(1.88-2.70) | <0.001 |
| Q1 | Reference | - | Reference | - | Reference | - |
| Q2 | 3.55(2.87-4.38) | <0.001 | 2.83(2.26-3.55) | <0.001 | 2.84(2.26-3.55) | <0.001 |
| Q3 | 5.88(4.64-7.46) | <0.001 | 4.88(3.78-6.30) | <0.001 | 4.87(3.77-6.29) | <0.001 |
| Q4 | 9.31(7.49-11.58) | <0.001 | 8.96(7.17-11.20) | <0.001 | 8.95(7.15-11.20) | <0.001 |
| *P* for trend | <0.001 |  | <0.001 |  | <0.001 |  |
| VAI |  |  |  |  |  |  |
| Per-SD increase | 1.76(1.46-2.11) | <0.001 | 1.81(1.48-2.21) | <0.001 | 1.81(1.48-2.21) | <0.001 |
| Q1 | Reference | - | Reference | - | Reference | - |
| Q2 | 2.27(1.76-2.93) | <0.001 | 2.12(1.65-2.72) | <0.001 | 2.12(1.65-2.72) | <0.001 |
| Q3 | 4.53(3.54-5.78) | <0.001 | 4.37(3.39-5.63) | <0.001 | 4.37(3.39-5.63) | <0.001 |
| Q4 | 6.01(4.71-7.69) | <0.001 | 6.32(4.97-8.04) | <0.001 | 6.31(4.94-8.06) | <0.001 |
| *P* for trend | <0.001 |  | <0.001 |  | <0.001 |  |
| CMI |  |  |  |  |  |  |
| Per-SD increase | 1.75(1.44-2.12) | <0.001 | 1.95(1.56-2.44) | <0.001 | 1.94(1.55-2.44) | <0.001 |
| Q1 | Reference | - | Reference | - | Reference | - |
| Q2 | 1.86(1.42-2.43) | <0.001 | 1.63(1.24-2.13) | <0.001 | 1.63(1.25-2.13) | <0.001 |
| Q3 | 3.49(2.73-4.47) | <0.001 | 3.34(2.60-4.29) | <0.001 | 3.33(2.59-4.29) | <0.001 |
| Q4 | 5.34(4.14-6.87) | <0.001 | 6.45(4.98-8.35) | <0.001 | 6.43(4.94-8.36) | <0.001 |
| *P* for trend | <0.001 |  | <0.001 |  | <0.001 |  |
| AIP |  |  |  |  |  |  |
| Per-SD increase | 1.75(1.61-1.91) | <0.001 | 1.96(1.78-2.15) | <0.001 | 1.96(1.78-2.15) | <0.001 |
| Q1 | Reference | - | Reference | - | Reference | - |
| Q2 | 1.87(1.45-2.40) | <0.001 | 1.81(1.42-2.31) | <0.001 | 1.81(1.42-2.31) | <0.001 |
| Q3 | 3.58(2.75-4.66) | <0.001 | 3.72(2.87-4.82) | <0.001 | 3.71(2.86-4.82) | <0.001 |
| Q4 | 4.56(3.62-5.73) | <0.001 | 5.80(4.64-7.26) | <0.001 | 5.79(4.62-7.27) | <0.001 |
| *P* for trend | <0.001 |  | <0.001 |  | <0.001 |  |
| TG/HDL-C |  |  |  |  |  |  |
| Per-SD increase | 1.56(1.30-1.86) | <0.001 | 1.73(1.39-2.15) | <0.001 | 1.73(1.39-2.15) | <0.001 |
| Q1 | Reference | - | Reference | - | Reference | - |
| Q2 | 1.88(1.46-2.41) | <0.001 | 1.81(1.42-2.31) | <0.001 | 1.81(1.43-2.31) | <0.001 |
| Q3 | 3.57(2.74-4.64) | <0.001 | 3.70(2.85-4.80) | <0.001 | 3.69(2.84-4.80) | <0.001 |
| Q4 | 4.59(3.65-5.77) | <0.001 | 5.84(4.67-7.31) | <0.001 | 5.83(4.65-7.32) | <0.001 |
| *P* for trend | <0.001 |  | <0.001 |  | <0.001 |  |
| METS-IR |  |  |  |  |  |  |
| Per-SD increase | 1.62(1.52-1.73) | <0.001 | 1.97(1.82-2.14) | <0.001 | 1.98(1.82-2.15) | <0.001 |
| Q1 | Reference | - | Reference | - | Reference | - |
| Q2 | 2.00(1.64-2.44) | <0.001 | 1.76(1.45-2.14) | <0.001 | 1.76(1.45-2.14) | <0.001 |
| Q3 | 2.42(1.98-2.95) | <0.001 | 2.68(2.12-3.39) | <0.001 | 2.68(2.12-3.38) | <0.001 |
| Q4 | 3.57(2.96-4.31) | <0.001 | 5.16(4.15-6.43) | <0.001 | 5.20(4.18-6.48) | <0.001 |
| *P* for trend | <0.001 |  | <0.001 |  | <0.001 |  |
| HOMA-IR |  |  |  |  |  |  |
| Per-SD increase | 2.73(2.22-3.36) | <0.001 | 3.60(2.91-4.46) | <0.001 | 3.61(2.91-4.48) | <0.001 |
| Q1 | Reference | - | Reference | - | Reference | - |
| Q2 | 1.80(1.52-2.12) | <0.001 | 1.76(1.46-2.11) | <0.001 | 1.76(1.47-2.12) | <0.001 |
| Q3 | 2.95(2.30-3.77) | <0.001 | 3.12(2.40-4.07) | <0.001 | 3.13(2.40-4.09) | <0.001 |
| Q4 | 3.07(2.47-3.80) | <0.001 | 3.91(3.13-4.89) | <0.001 | 3.92(3.14-4.90) | <0.001 |
| *P* for trend | <0.001 |  | <0.001 |  | <0.001 |  |
| TyG |  |  |  |  |  |  |
| Per-SD increase | 2.21(1.99-2.46) | <0.001 | 2.23(2.00-2.50) | <0.001 | 2.23(1.99-2.49) | <0.001 |
| Q1 | Reference | - | Reference | - | Reference | - |
| Q2 | 3.24(2.63-4.00) | <0.001 | 2.68(2.15-3.35) | <0.001 | 2.69(2.15-3.36) | <0.001 |
| Q3 | 5.86(4.50-7.63) | <0.001 | 4.97(3.78-6.54) | <0.001 | 4.97(3.78-6.53) | <0.001 |
| Q4 | 7.86(6.17-10.01) | <0.001 | 7.82(6.07-10.06) | <0.001 | 7.80(6.05-10.04) | <0.001 |
| *P* for trend | <0.001 |  | <0.001 |  | <0.001 |  |
| TyG-BMI |  |  |  |  |  |  |
| Per-SD increase | 2.13(1.97-2.30) | <0.001 | 2.37(2.16-2.60) | <0.001 | 2.37(2.16-2.61) | <0.001 |
| Q1 | Reference | - | Reference | - | Reference | - |
| Q2 | 2.98(2.43-3.64) | <0.001 | 2.44(1.98-3.01) | <0.001 | 2.44(1.98-3.01) | <0.001 |
| Q3 | 4.13(3.36-5.09) | <0.001 | 3.84(3.01-4.90) | <0.001 | 3.83(3.00-4.87) | <0.001 |
| Q4 | 7.37(5.87-9.25) | <0.001 | 8.62(6.79-10.95) | <0.001 | 8.65(6.81-11.00) | <0.001 |
| *P* for trend | <0.001 |  | <0.001 |  | <0.001 |  |
| TyG-WC |  |  |  |  |  |  |
| Per-SD increase | 2.15(2.00-2.31) | <0.001 | 2.29(2.11-2.50) | <0.001 | 2.30(2.11-2.50) | <0.001 |
| Q1 | Reference | - | Reference | - | Reference | - |
| Q2 | 2.80(2.23-3.52) | <0.001 | 2.23(1.74-2.86) | <0.001 | 2.23(1.74-2.86) | <0.001 |
| Q3 | 5.12(4.11-6.39) | <0.001 | 4.40(3.42-5.66) | <0.001 | 4.39(3.41-5.66) | <0.001 |
| Q4 | 6.36(4.99-8.10) | <0.001 | 6.58(5.06-8.56) | <0.001 | 6.59(5.06-8.58) | <0.001 |
| *P* for trend | <0.001 |  | <0.001 |  | <0.001 |  |
| TyG-WHtR |  |  |  |  |  |  |
| Per-SD increase | 2.23(2.01-2.47) | <0.001 | 2.25(2.01-2.51) | <0.001 | 2.24(2.00-2.51) | <0.001 |
| Q1 | Reference | - | Reference | - | Reference | - |
| Q2 | 3.25(2.66-3.98) | <0.001 | 2.70(2.18-3.35) | <0.001 | 2.71(2.18-3.35) | <0.001 |
| Q3 | 5.97(4.61-7.73) | <0.001 | 5.09(3.89-6.67) | <0.001 | 5.08(3.88-6.65) | <0.001 |
| Q4 | 7.87(6.16-10.05) | <0.001 | 7.75(6.00-10.00) | <0.001 | 7.73(5.98-9.99) | <0.001 |
| *P* for trend | <0.001 |  | <0.001 |  | <0.001 |  |
| TyG-ABSI |  |  |  |  |  |  |
| Per-SD increase | 2.54(2.30-2.80) | <0.001 | 2.11(1.89-2.36) | <0.001 | 2.11(1.89-2.36) | <0.001 |
| Q1 | Reference | - | Reference | - | Reference | - |
| Q2 | 2.95(2.39-3.65) | <0.001 | 2.42(1.92-3.05) | <0.001 | 2.42(1.91-3.05) | <0.001 |
| Q3 | 5.72(4.67-7.01) | <0.001 | 4.10(3.21-5.25) | <0.001 | 4.10(3.20-5.25) | <0.001 |
| Q4 | 8.42(6.74-10.53) | <0.001 | 5.21(3.99-6.79) | <0.001 | 5.17(3.95-6.78) | <0.001 |
| *P* for trend | <0.001 |  | <0.001 |  | <0.001 |  |

Model 1: Crude (unadjusted).

Model 2: Adjusted for age, sex, race, PIR, marital status, education levels.

Model 3: Model 2 + adjustments for tobacco and alcohol use.

**Supplementary Table 6**. **Sensitivity analysis of different surrogate IR indexes on the risk of CMM in participants without hyperlipidemia (N=3,454).**

|  | Model 1 | | Model 2 | | Model 3 | |
| --- | --- | --- | --- | --- | --- | --- |
|  | OR (95% CI) | *p* Value | OR (95% CI) | *p* Value | OR (95% CI) | *p* Value |
| LAP |  |  |  |  |  |  |
| Per-SD increase | 1.87(1.64-2.13) | <0.001 | 1.71(1.47-1.99) | <0.001 | 1.71(1.47-1.99) | <0.001 |
| Q1 | Reference | - | Reference | - | Reference | - |
| Q2 | 2.86(1.63-5.00) | <0.001 | 1.45(0.77-2.74) | 0.252 | 1.45(0.77-2.74) | 0.252 |
| Q3 | 5.45(3.55-8.88) | <0.001 | 2.31(1.33-4.02) | 0.003 | 2.31(1.33-4.02) | 0.003 |
| Q4 | 9.70(5.99-15.70) | <0.001 | 4.06(2.34-7.05) | <0.001 | 4.06(2.34-7.05) | <0.001 |
| *P* for trend | <0.001 |  | <0.001 |  | <0.001 |  |
| VAI |  |  |  |  |  |  |
| Per-SD increase | 1.93(1.71-2.16) | <0.001 | 1.74(1.50-2.01) | <0.001 | 1.74(1.50-2.01) | <0.001 |
| Q1 | Reference | - | Reference | - | Reference | - |
| Q2 | 2.27(1.41-3.67) | <0.001 | 1.73(1.00-2.98) | 0.05 | 1.73(1.00-2.98) | 0.05 |
| Q3 | 3.72(2.44-5.67) | <0.001 | 2.38(1.44-3.95) | <0.001 | 2.38(1.44-3.95) | <0.001 |
| Q4 | 6.95(4.62-10.46) | <0.001 | 4.53(2.80-7.33) | <0.001 | 4.53(2.80-7.33) | <0.001 |
| *P* for trend | <0.001 |  | <0.001 |  | <0.001 |  |
| CMI |  |  |  |  |  |  |
| Per-SD increase | 1.81(1.60-2.04) | <0.001 | 1.78(1.54-2.06) | <0.001 | 1.78(1.54-2.06) | <0.001 |
| Q1 | Reference | - | Reference | - | Reference | - |
| Q2 | 2.22(1.40-3.54) | <0.001 | 1.84(1.11-3.07) | 0.019 | 1.84(1.11-3.07) | 0.019 |
| Q3 | 4.59(3.01-6.99) | <0.001 | 2.94(1.85-4.67) | <0.001 | 2.94(1.85-4.67) | <0.001 |
| Q4 | 7.91(5.41-11.57) | <0.001 | 4.99(3.20-7.80) | <0.001 | 4.99(3.20-7.80) | <0.001 |
| *P* for trend | <0.001 |  | <0.001 |  | <0.001 |  |
| AIP |  |  |  |  |  |  |
| Per-SD increase | 1.85(1.59-2.15) | <0.001 | 1.74(1.41-2.14) | <0.001 | 1.74(1.42-2.14) | <0.001 |
| Q1 | Reference | - | Reference | - | Reference | - |
| Q2 | 1.68(1.13-2.49) | 0.010 | 1.74(1.13-2.67) | 0.012 | 1.76(1.15-2.71) | 0.010 |
| Q3 | 2.80(1.89-4.15) | <0.001 | 2.39(1.54-3.72) | <0.001 | 2.41(1.55-3.76) | <0.001 |
| Q4 | 5.23(3.77-7.24) | <0.001 | 4.42(3.03-6.45) | <0.001 | 4.46(3.05-6.52) | <0.001 |
| *P* for trend | <0.001 |  | <0.001 |  | <0.001 |  |
| TG/HDL-C |  |  |  |  |  |  |
| Per-SD increase | 1.70(1.51-1.91) | <0.001 | 1.73(1.48-2.03) | <0.001 | 1.73(1.48-2.03) | <0.001 |
| Q1 | Reference | - | Reference | - | Reference | - |
| Q2 | 1.68(1.13-2.50) | 0.011 | 1.78(1.16-2.73) | 0.009 | 1.78(1.16-2.73) | 0.009 |
| Q3 | 2.77(1.87-4.12) | <0.001 | 2.42(1.55-3.78) | <0.001 | 2.42(1.55-3.78) | <0.001 |
| Q4 | 5.20(3.75-7.21) | <0.001 | 4.42(3.03-6.46) | <0.001 | 4.42(3.03-6.46) | <0.001 |
| *P* for trend | <0.001 |  | <0.001 |  | <0.001 |  |
| METS-IR |  |  |  |  |  |  |
| Per-SD increase | 1.69(1.50-1.90) | <0.001 | 1.80(1.56-2.07) | <0.001 | 1.80(1.56-2.07) | <0.001 |
| Q1 | Reference | - | Reference | - | Reference | - |
| Q2 | 1.08(0.72-1.62) | 0.722 | 0.79(0.50-1.25) | 0.305 | 0.79(0.50-1.25) | 0.305 |
| Q3 | 2.69(1.76-4.09) | <0.001 | 1.52(0.97-2.37) | 0.069 | 1.52(0.97-2.37) | 0.069 |
| Q4 | 4.16(2.79-6.21) | <0.001 | 3.50(2.17-5.63) | <0.001 | 3.50(2.17-5.63) | <0.001 |
| *P* for trend | <0.001 |  | <0.001 |  | <0.001 |  |
| HOMA-IR |  |  |  |  |  |  |
| Per-SD increase | 2.74(2.21-3.41) | <0.001 | 2.56(2.02-3.25) | <0.001 | 2.56(2.02-3.25) | <0.001 |
| Q1 | Reference | - | Reference | - | Reference | - |
| Q2 | 1.26(0.80-1.99) | 0.321 | 1.12(0.67-1.86) | 0.666 | 1.12(0.67-1.86) | 0.666 |
| Q3 | 2.35(1.52-3.64) | <0.001 | 1.71(1.05-2.78) | 0.032 | 1.71(1.05-2.78) | 0.032 |
| Q4 | 5.71(3.87-8.42) | <0.001 | 4.17(2.69-6.46) | <0.001 | 4.17(2.69-6.46) | <0.001 |
| *P* for trend | <0.001 |  | <0.001 |  | <0.001 |  |
| TyG |  |  |  |  |  |  |
| Per-SD increase | 3.05(2.52-3.69) | <0.001 | 2.50(1.98-3.16) | <0.001 | 2.50(1.98-3.16) | <0.001 |
| Q1 | Reference | - | Reference | - | Reference | - |
| Q2 | 1.72(1.06-2.79) | 0.029 | 1.26(0.73-2.19) | 0.403 | 1.26(0.73-2.19) | 0.403 |
| Q3 | 3.41(2.17-5.36) | <0.001 | 2.08(1.24-3.49) | 0.006 | 2.08(1.24-3.49) | 0.006 |
| Q4 | 11.85(7.72-18.18) | <0.001 | 6.53(3.93-10.87) | <0.001 | 6.53(3.93-10.87) | <0.001 |
| *P* for trend | <0.001 |  | <0.001 |  | <0.001 |  |
| TyG-BMI |  |  |  |  |  |  |
| Per-SD increase | 1.74(1.54-1.98) | <0.001 | 1.78(1.54-2.05) | <0.001 | 1.77(1.53-2.05) | <0.001 |
| Q1 | Reference | - | Reference | - | Reference | - |
| Q2 | 1.22(0.78-1.89) | 0.381 | 0.80(0.50-1.26) | 0.328 | 0.80(0.51-1.25) | 0.326 |
| Q3 | 2.53(1.73-3.72) | <0.001 | 1.28(0.82-2.01) | 0.279 | 1.28(0.82-2.00) | 0.282 |
| Q4 | 4.34(2.93-6.41) | <0.001 | 3.08(1.92-4.94) | <0.001 | 3.07(1.91-4.91) | <0.001 |
| *P* for trend | <0.001 |  | <0.001 |  | <0.001 |  |
| TyG-WC |  |  |  |  |  |  |
| Per-SD increase | 2.14(1.86-2.45) | <0.001 | 2.01(1.71-2.36) | <0.001 | 2.00(1.70-2.36) | <0.001 |
| Q1 | Reference | - | Reference | - | Reference | - |
| Q2 | 2.44(1.38-4.32) | 0.002 | 1.43(0.77-2.63) | 0.254 | 1.43(0.78-2.63) | 0.240 |
| Q3 | 5.24(2.95-9.34) | <0.001 | 2.31(1.26-4.23) | 0.007 | 2.31(1.27-4.22) | 0.007 |
| Q4 | 10.27(5.72-18.44) | <0.001 | 4.61(2.44-8.71) | <0.001 | 4.60(2.43-8.68) | <0.001 |
| *P* for trend | <0.001 |  | <0.001 |  | <0.001 |  |
| TyG-WHtR |  |  |  |  |  |  |
| Per-SD increase | 2.38(2.06-2.74) | <0.001 | 2.04(1.75-2.39) | <0.001 | 2.04(1.74-2.39) | <0.001 |
| Q1 | Reference | - | Reference | - | Reference | - |
| Q2 | 3.56(1.94-6.55) | <0.001 | 1.62(0.85-3.08) | 0.141 | 1.62(0.86-3.06) | 0.135 |
| Q3 | 8.01(4.20-15.26) | <0.001 | 2.73(1.43-5.23) | 0.003 | 2.75(1.44-5.25) | 0.002 |
| Q4 | 15.80(8.33-29.97) | <0.001 | 5.47(2.75-10.91) | <0.001 | 5.46(2.75-10.84) | <0.001 |
| *P* for trend | <0.001 |  | <0.001 |  | <0.001 |  |
| TyG-ABSI |  |  |  |  |  |  |
| Per-SD increase | 3.56(3.10-4.08) | <0.001 | 2.40(1.96-2.93) | <0.001 | 2.40(1.96-2.93) | <0.001 |
| Q1 | Reference | - | Reference | - | Reference | - |
| Q2 | 1.79(1.00-3.20) | 0.052 | 1.12(0.57-2.18) | 0.744 | 1.12(0.57-2.18) | 0.744 |
| Q3 | 5.88(3.25-10.63) | <0.001 | 2.63(1.29-5.36) | 0.008 | 2.63(1.29-5.36) | 0.008 |
| Q4 | 20.36(12.46-33.28) | <0.001 | 6.12(3.16-11.86) | <0.001 | 6.12(3.16-11.86) | <0.001 |
| *P* for trend | <0.001 |  | <0.001 |  | <0.001 |  |

Model 1: Crude (unadjusted).

Model 2: Adjusted for age, sex, race, PIR, marital status, education levels.

Model 3: Model 2 + adjustments for tobacco and alcohol use.

**Supplementary Table 7**. **Sensitivity analysis of different surrogate IR indexes with CMM after additional adjustments for BMI and waist circumference.**

|  | Model 1 | | Model 3 | | Model 4 | |
| --- | --- | --- | --- | --- | --- | --- |
|  | OR (95% CI) | *p* Value | OR (95% CI) | *p* Value | OR (95% CI) | *p* Value |
| LAP |  |  |  |  |  |  |
| Per-SD increase | 2.99(2.50-3.57) | <0.001 | 2.97(2.49-3.53) | <0.001 | 2.25(1.81-2.80) | <0.001 |
| Q1 | Reference | - | Reference | - | Reference | - |
| Q2 | 3.26(3.00-4.38) | <0.001 | 2.86(2.30-3.55) | <0.001 | 2.81(2.23-3.53) | <0.001 |
| Q3 | 6.70(5.54-8.11) | <0.001 | 5.37(4.35-6.63) | <0.001 | 4.93(3.93-6.20) | <0.001 |
| Q4 | 13.89(11.57-16.67) | <0.001 | 13.26(10.94-16.06) | <0.001 | 10.90(8.43-14.09) | <0.001 |
| *P* for trend | <0.001 |  | <0.001 |  | <0.001 |  |
| VAI |  |  |  |  |  |  |
| Per-SD increase | 2.41(2.01-2.90) | <0.001 | 2.41(1.99-2.93) | <0.001 | 1.99(1.65-2.41) | <0.001 |
| Q1 | Reference | - | Reference | - | Reference | - |
| Q2 | 2.35(1.91-2.90) | <0.001 | 2.16(1.75-2.67) | <0.001 | 1.82(1.45-2.28) | <0.001 |
| Q3 | 5.07(4.22, 6.10) | <0.001 | 4.93(4.05-6.00) | <0.001 | 3.72(3.05, 4.55) | <0.001 |
| Q4 | 9.17(7.58-11.09) | <0.001 | 9.78(8.06-11.87) | <0.001 | 6.92(5.64-8.49) | <0.001 |
| *P* for trend | <0.001 |  | <0.001 |  | <0.001 |  |
| CMI |  |  |  |  |  |  |
| Per-SD increase | 2.49(2.04-3.04) | <0.001 | 2.66(2.13-3.33) | <0.001 | 2.01(1.61-2.51) | <0.001 |
| Q1 | Reference | - | Reference | - | Reference | - |
| Q2 | 2.23(1.82-2.73) | <0.001 | 1.92(1.54-2.38) | <0.001 | 1.58(1.27-1.98) | <0.001 |
| Q3 | 4.55(3.69-5.61) | <0.001 | 4.31(3.50-5.31) | <0.001 | 3.14(2.52-3.91) | <0.001 |
| Q4 | 8.84(7.20-10.85) | <0.001 | 10.49(8.44-13.05) | <0.001 | 6.81(5.37-8.63) | <0.001 |
| *P* for trend | <0.001 |  | <0.001 |  | <0.001 |  |
| AIP |  |  |  |  |  |  |
| Per-SD increase | 2.10(1.96-2.26) | <0.001 | 2.35 (2.17–2.55) | <0.001 | 2.09(1.92-2.28) | <0.001 |
| Q1 | Reference | - | Reference | - | Reference | - |
| Q2 | 2.18(1.78-2.66) | <0.001 | 2.11(1.73-2.57) | <0.001 | 1.81(1.46-2.25) | <0.001 |
| Q3 | 4.31(3.49-5.32) | <0.001 | 4.48(3.63-5.53) | <0.001 | 3.40(2.74-4.22) | <0.001 |
| Q4 | 7.25(6.02-8.73) | <0.001 | 9.29(7.74-11.16) | <0.001 | 6.77(5.56-8.24) | <0.001 |
| *P* for trend | <0.001 |  | <0.001 |  | <0.001 |  |
| TG/HDL-C |  |  |  |  |  |  |
| Per-SD increase | 2.10(1.74-2.52) | <0.001 | 2.27(1.83-2.82) | <0.001 | 1.92(1.55-2.38) | <0.001 |
| Q1 | Reference | - | Reference | - | Reference | - |
| Q2 | 2.21(1.80-2.71) | <0.001 | 2.13(1.75-2.61) | <0.001 | 1.83(1.47-2.27) | <0.001 |
| Q3 | 4.33(3.51-5.35) | <0.001 | 4.50(3.65-5.55) | <0.001 | 3.41(2.75-4.22) | <0.001 |
| Q4 | 7.35(6.09-8.87) | <0.001 | 9.42(7.83-11.33) | <0.001 | 6.85(5.61-8.35) | <0.001 |
| *P* for trend | <0.001 |  | <0.001 |  | <0.001 |  |
| METS-IR |  |  |  |  |  |  |
| Per-SD increase | 1.99(1.86-2.12) | <0.001 | 2.39(2.22-2.57) | <0.001 | 3.69(3.11-4.38) | <0.001 |
| Q1 | Reference | - | Reference | - | Reference | - |
| Q2 | 2.24(1.86-2.70) | <0.001 | 1.89(1.57-2.28) | <0.001 | 2.38(1.89-2.99) | <0.001 |
| Q3 | 3.10(2.57-3.73) | <0.001 | 3.24(2.62-4.00) | <0.001 | 5.25(3.98-6.93) | <0.001 |
| Q4 | 6.13(5.13-7.32) | <0.001 | 8.75(7.16-10.69) | <0.001 | 14.21(9.42-21.44) | <0.001 |
| *P* for trend | <0.001 |  | <0.001 |  | <0.001 |  |
| HOMA-IR |  |  |  |  |  |  |
| Per-SD increase | 5.81(4.71-7.16) | <0.001 | 6.83(5.53-8.44) | <0.001 | 4.21(3.45-5.12) | <0.001 |
| Q1 | Reference | - | Reference | - | Reference | - |
| Q2 | 1.87(1.57-2.23) | <0.001 | 1.78(1.48-2.14) | <0.001 | 1.52(1.26-1.83) | <0.001 |
| Q3 | 3.52(2.82-4.41) | <0.001 | 3.60(2.82-4.60) | <0.001 | 2.68(2.08-3.46) | <0.001 |
| Q4 | 7.16(5.92-8.66) | <0.001 | 8.80(7.31-10.60) | <0.001 | 5.44(4.42-6.68) | <0.001 |
| *P* for trend | <0.001 |  | <0.001 |  | <0.001 |  |
| TyG |  |  |  |  |  |  |
| Per-SD increase | 3.00(2.74-3.28) | <0.001 | 3.01(2.73-3.31) | <0.001 | 2.69(2.43-2.97) | <0.001 |
| Q1 | Reference | - | Reference | - | Reference | - |
| Q2 | 3.30(2.74-3.96) | <0.001 | 2.70(2.22-3.28) | <0.001 | 2.38(1.96-2.87) | <0.001 |
| Q3 | 6.58(5.26-8.24) | <0.001 | 5.59(4.40-7.12) | <0.001 | 4.42(3.51-5.56) | <0.001 |
| Q4 | 16.27(13.22-20.03) | <0.001 | 15.48(12.49-19.20) | <0.001 | 11.65(9.32-14.56) | <0.001 |
| *P* for trend | <0.001 |  | <0.001 |  | <0.001 |  |
| TyG-BMI |  |  |  |  |  |  |
| Per-SD increase | 2.09(1.96-2.23) | <0.001 | 2.37(2.20-2.55) | <0.001 | 4.18(3.43-5.10) | <0.001 |
| Q1 | Reference | - | Reference | - | Reference | - |
| Q2 | 2.78(2.24-3.45) | <0.001 | 2.19(1.75-2.74) | <0.001 | 3.93(2.92-5.30) | <0.001 |
| Q3 | 4.10(3.39-4.97) | <0.001 | 3.89(3.10-4.90) | <0.001 | 12.02(8.43-17.13) | <0.001 |
| Q4 | 7.61(6.36-9.11) | <0.001 | 9.33(7.60-11.47) | <0.001 | 31.37(20.64-47.67) | <0.001 |
| *P* for trend | <0.001 |  | <0.001 |  | <0.001 |  |
| TyG-WC |  |  |  |  |  |  |
| Per-SD increase | 2.56(2.40-2.73) | <0.001 | 2.69(2.50-2.89) | <0.001 | 11.43(8.89-14.69) | <0.001 |
| Q1 | Reference | - | Reference | - | Reference | - |
| Q2 | 2.80(2.23-3.51) | <0.001 | 2.18(1.68-2.84) | <0.001 | 2.88(2.12-3.93) | <0.001 |
| Q3 | 5.29(4.35-6.43) | <0.001 | 4.44(3.55-5.55) | <0.001 | 7.20(4.97-10.43) | <0.001 |
| Q4 | 10.58(8.53-13.11) | <0.001 | 10.68(8.46-13.50) | <0.001 | 20.34(13.19-31.36) | <0.001 |
| *P* for trend | <0.001 |  | <0.001 |  | <0.001 |  |
| TyG-WHtR |  |  |  |  |  |  |
| Per-SD increase | 2.82(2.63-3.03) | <0.001 | 2.70(2.51-2.91) | <0.001 | 6.45(5.23-7.95) | <0.001 |
| Q1 | Reference | - | Reference | - | Reference | - |
| Q2 | 3.62(3.03-4.33) | <0.001 | 2.57(2.08-3.18) | <0.001 | 3.36(2.64-4.28) | <0.001 |
| Q3 | 6.50(5.37-7.86) | <0.001 | 4.71(3.85-5.77) | <0.001 | 7.51(5.60-10.07) | <0.001 |
| Q4 | 14.88(12.12-18.27) | <0.001 | 11.85(9.51-14.75) | <0.001 | 21.17(13.62-32.91) | <0.001 |
| *P* for trend | <0.001 |  | <0.001 |  | <0.001 |  |
| TyG-ABSI |  |  |  |  |  |  |
| Per-SD increase | 3.34(3.07-3.63) | <0.001 | 2.89(2.63-3.17) | <0.001 | 2.51(2.28-2.77) | <0.001 |
| Q1 | Reference | - | Reference | - | Reference | - |
| Q2 | 3.01(2.51-3.63) | <0.001 | 2.51(2.04-3.11) | <0.001 | 2.08(1.67-2.60) | <0.001 |
| Q3 | 6.69(5.63-7.96) | <0.001 | 4.93(3.98-6.10) | <0.001 | 3.59(2.87-4.49) | <0.001 |
| Q4 | 16.95(14.15-20.31) | <0.001 | 11.02(8.86-13.71) | <0.001 | 7.41(5.88-9.35) | <0.001 |
| *P* for trend | <0.001 |  | <0.001 |  | <0.001 |  |

Model 1: Crude (unadjusted).

Model 3: Adjusted for age, sex, race, PIR, marital status, education levels, tobacco and alcohol use.

Model 4: Model 3 + adjustments for BMI and waist circumference.

**Supplementary Table 8. Interaction analysis of education levels, marital status and PIR on the association between IR indices and CMM.**

|  | P for interaction | | |
| --- | --- | --- | --- |
|  | Education levels | Marital status | PIR |
| LAP | 0.754 | 0.085 | 0.893 |
| VAI | 0.263 | 0.045 | 0.735 |
| CMI | 0.665 | 0.006 | 0.559 |
| AIP | 0.103 | 0.171 | 0.533 |
| TG/HDL-C | 0.520 | 0.004 | 0.617 |
| METS-IR | 0.186 | 0.872 | 0.147 |
| HOMA-IR | 0.134 | 0.847 | <0.001 |
| TyG | 0.129 | 0.154 | 0.943 |
| TyG-BMI | 0.232 | 0.801 | 0.242 |
| TyG-WC | 0.079 | 0.683 | 0.495 |
| TyG-WHtR | 0.230 | 0.552 | 0.636 |
| TyG-ABSI | 0.044 | 0.259 | 0.857 |

**Supplementary Table 9**. **The proportion of missing data for key variables.**

| Variables | Missing percentage, n (%) |
| --- | --- |
| Age | 0 (0%) |
| Sex | 0 (0%) |
| Race | 0 (0%) |
| Education levels | 52698 (45.09%) |
| Marital status | 46131 (39.47%) |
| PIR | 11397 (9.75%) |
| Tobacco use | 51270 (43.87%) |
| Alcohol use | 90019 (77.02%) |
| BMI | 15940 (13.64%) |
| Height | 15480 (13.24%) |
| Waist Circumference | 19603 (16.77%) |
| Triglycerides | 38194 (32.68%) |
| Glucose | 37532 (32.11%) |
| HDL-C | 30042 (25.7%) |
| HbA1c | 38143 (32.64%) |
| CAD | 0 (0%) |
| Hypertension (HTN) | 0 (0%) |
| Diabetes (DM) | 0 (0%) |
| Stroke | 0 (0%) |
| Dyslipidemia | 0 (0%) |

**
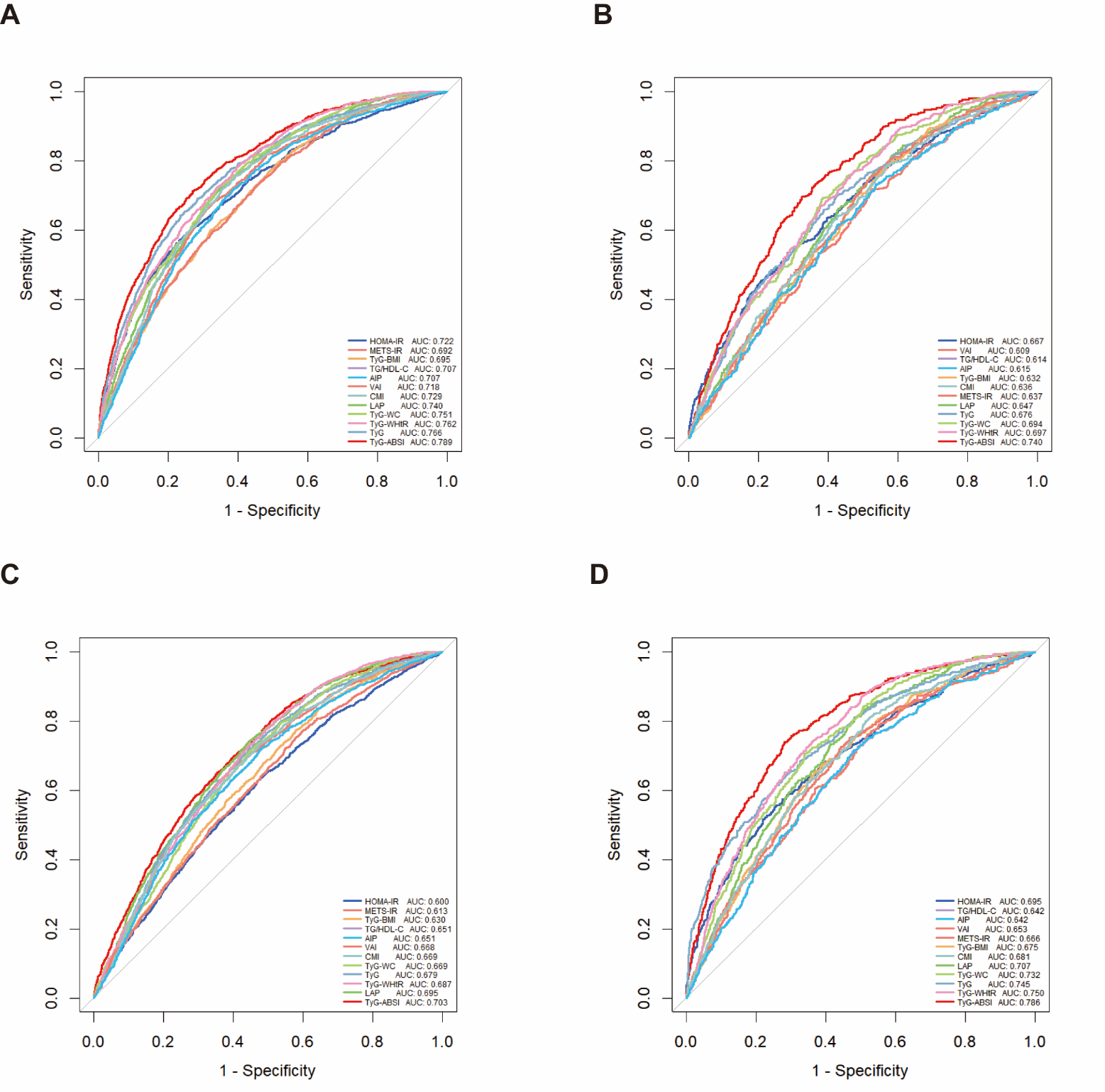
**

**Supplementary Fig. 1. ROC curves for the association between diverse IR indexes and CMM across four sensitivity analysis populations.**
